# Human α-synuclein overexpression in mouse serotonin neurons triggers a depressive-like phenotype. Rescue by oligonucleotide therapy

**DOI:** 10.1038/s41398-022-01842-z

**Published:** 2022-02-24

**Authors:** Lluis Miquel-Rio, Diana Alarcón-Arís, María Torres-López, Valentín Cóppola-Segovia, Rubén Pavia-Collado, Verónica Paz, Esther Ruiz-Bronchal, Leticia Campa, Carme Casal, Andrés Montefeltro, Miquel Vila, Francesc Artigas, Raquel Revilla, Analia Bortolozzi

**Affiliations:** 1 Institut d’Investigacions Biomèdiques de Barcelona (IIBB), Spanish National Research Council (CSIC), 08036 Barcelona, Spain; 2 Institut d’Investigacions Biomèdiques August Pi i Sunyer (IDIBAPS), 08036 Barcelona, Spain; 3 Centro de Investigación Biomédica en Red de Salud Mental (CIBERSAM), ISCIII, 28029 Madrid, Spain; 4 Universitat de Barcelona (UB), 08036 Barcelona, Spain; 5 Federal University of Paraná (UFPR), Curitiba, 81531-980 Brazil; 6 Lingea MC, AD400 Erts, Andorra; 7 Neurodegenerative Diseases Research Group, Vall d’Hebron Research Institute, 08035 Barcelona, Spain; 8 Centro de Investigación Biomédica en Red de Enfermedades Neurodegenerativas (CIBERNED), ISCIII, 28031 Madrid, Spain; 9 Catalan Institution for Research and Advanced Studies (ICREA), 08010 Barcelona, Spain

**Keywords:** Psychiatric disorders, Neuroscience

## Abstract

Anxiety and depression affect 35–50% of patients with Parkinson’s disease (PD), often precede the onset of motor symptoms, and have a negative impact on their quality of life. Dysfunction of the serotonergic (5-HT) system, which regulates mood and emotional pathways, occurs during the premotor phase of PD and contributes to a variety of non-motor symptoms. Furthermore, α-synuclein (α-Syn) aggregates were identified in raphe nuclei in the early stages of the disease. However, there are very few animal models of PD-related neuropsychiatric disorders. Here, we develop a new mouse model of α-synucleinopathy in the 5-HT system that mimics prominent histopathological and neuropsychiatric features of human PD. We showed that adeno-associated virus (AAV5)-induced overexpression of wild-type human α-Syn (h-α-Syn) in raphe 5-HT neurons triggers progressive accumulation, phosphorylation, and aggregation of h-α-Syn protein in the 5-HT system. Specifically, AAV5-injected mice displayed axonal impairment in the output brain regions of raphe neurons, and deficits in brain-derived neurotrophic factor (BDNF) expression and 5-HT neurotransmission, resulting in a depressive-like phenotype. Intracerebroventricular treatment with an indatraline-conjugated antisense oligonucleotide (IND-ASO) for four weeks induced an effective and safe reduction of h-α-Syn synthesis in 5-HT neurons and its accumulation in the forebrain, alleviating early deficits of 5-HT function and improving the behavioural phenotype. Altogether, our findings show that α-synucleinopathy in 5-HT neurons negatively affects brain circuits that control mood and emotions, resembling the expression of neuropsychiatric symptoms occurring at the onset of PD. Early preservation of 5-HT function by reducing α-Syn synthesis/accumulation may alleviate PD-related depressive symptoms.

## Introduction

Parkinson’s disease (PD) is a progressive neurological disorder characterized by the neurodegeneration of dopamine (DA) neurons in the nigrostriatal pathway and the accumulation of α-synuclein (α-Syn) protein through the brain [1–3]. Under physiological conditions, α-Syn plays a key role in synaptic transmission, neuroplasticity, and monoamine homeostasis [4–9]. In PD brains, α-Syn undergoes conformational changes that render the protein prone to aggregation and accumulation promoting Lewy body (LB) pathology. Mutations in the *SNCA* gene, which encodes the α-Syn protein, can translate into α-Syn misfolding and aggregation, and early-onset forms of PD [10–12]. Furthermore, the identification of families with *SNCA* gene duplications or triplications strengthened the link between α-Syn and PD, which suggests that increasing concentrations of the wild-type α-Syn protein alone can cause the disease [13, 14].

Beyond the well-known role of nigrostriatal DA dysfunction in the pathophysiology of PD motor symptoms [15, 16], DA disruption of the mesocorticolimbic pathway is also involved in the occurrence of several non-motor manifestations such as apathy, fatigue, or impulse control disorders [17–20]. In addition, increasing lines of evidence support a specific causal role of serotonergic (5-HT) dysfunction in the pathogenesis of several PD symptoms, such as tremor and dyskinesia, but also anxiety, depression, anhedonia, cognitive decline, and hallucinations at early stages of the disease [21–27]. Indeed, anxiety and depression are the most prevalent neuropsychiatric symptom clusters in the PD population ranging from 35 to 50% [28–31]. Supporting a role for α-Syn in neuropsychiatric symptoms, neuropathological studies have shown the involvement of 5-HT neurons associated with the presence of LB pathology in the raphe nuclei in idiopathic PD, as well as in patients with triplication or mutation of *SNCA* gene [32–34]. Pioneering studies reported a loss of 5-HT neurons in the raphe nuclei of depressed PD patients with LB pathology [35–37], even reaching a 56% reduction [33]. More recently, in vivo neuroimaging studies in patients with idiopathic PD or A53T *SNCA* mutation carriers revealed an early progressive loss of 5-HT function, before nigrostriatal DA loss, highlighting the early role of 5-HT pathology in PD progression [38, 39].

Despite the high incidence of anxiety and depression in PD, there are still no established animal models for their study, as evidenced by the relatively sparse existing literature (e.g., only 75 publications reporting anxiety/depression-like effects in PD-like mouse models were found from 2010). Furthermore, most of the animal studies concerning anxiety and depression in PD focused primarily on DA pathways, which probably does not reflect the complexity underlying the occurrence of these symptoms in patients [40]. In fact, there is still a paucity of studies addressing the role of the other brain circuits in the early stages of the disease. Thus, reductions of 5-HT levels and 5-HT fiber density in the hippocampus (HPC), accompanied by an anxiety-like phenotype were reported in transgenic animal models overexpressing different forms of α-Syn [41, 42]. Likewise, a model of adeno-associated virus (AAV)-induced α-synucleinopathy selectively in 5-HT neurons of rats resulted in progressive degeneration of the 5-HT axon terminals in HPC, without loss of raphe 5-HT neurons [43]. Therefore, animal models involving an abnormal accumulation of α-Syn in raphe 5-HT neurons could help us to understand the relative role of the 5-HT system in non-motor PD symptoms. Indeed, although current treatments improve motor symptoms, treatments for anxiety/depression symptoms show minimal effectiveness, and no therapies can reduce or stop disease progression. Pathogenesis must be further explored, especially regarding the contribution of α-Syn pathology on anxiety and depression.

Here, we show that AAV-induced overexpression of wild-type human α-Syn (h-α-Syn) in raphe 5-HT neurons leads to (i) α-Syn pathology in raphe nuclei and efferent brain areas, (ii) brain functional deficits and, (iii) anxiety/depressive-like phenotype. This occurs within a temporal pattern that strikingly mirrors that observed in early-stage, idiopathic PD patients, supporting this model for preclinical testing of putative disease-modifying agents. Therefore, we examined the therapeutic benefit of a conjugated oligonucleotide targeting h-α-Syn selectively in 5-HT neurons [9, 44, 45] to attenuate the depressive-like phenotype induced by h-α-Syn overexpression.

## Materials and methods

All experimental procedures and data are included in the article and in Supplementary information. Detailed statistical analysis are shown in Supplementary Table 1. Materials and protocols are available upon request from the corresponding author (A.B.).

## Results

### Characterization of the mouse model with h-α-Syn overexpression in raphe 5-HT neurons

**Fig. 1:**
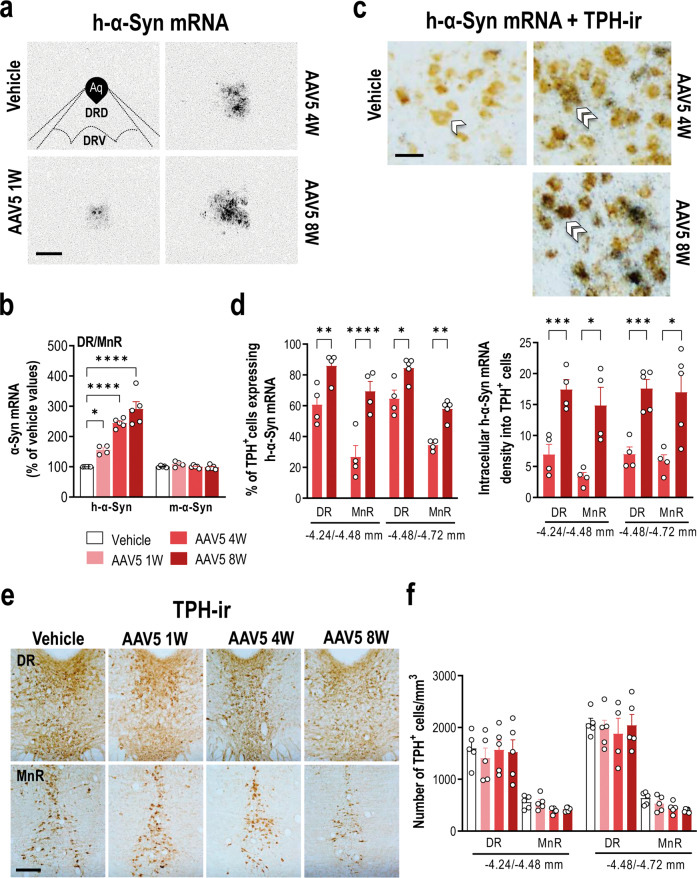
Overexpression of h-α-Syn transgene in raphe 5-HT neurons of mice. Mice received 1 μl AAV5 construct containing a chicken-β-actin promoter to drive expression of h-α-Syn or vehicle into raphe nuclei and euthanized at 1, 4, and 8 weeks (W) post-injection. **a** Coronal brain sections showing h-α-Syn mRNA levels in the raphe nuclei assessed by in situ hybridization. Scale bar: 500 μm. **b** Progressive increases of h-α-Syn mRNA expression in AAV5-injected mice compared to vehicle-injected mice. No differences were detected for murine α-Syn (m-α-Syn) mRNA expression in the raphe nuclei. **c** Photomicrographs showing TPH-positive neurons expressing h-α-Syn mRNA (^33^P-oligonucleotide silver grains) in the dorsal raphe nucleus (DR) of vehicle- and AAV5-injected mice at 4 and 8 W later. Scale bar: 20 μm. Single and double white arrowheads show TPH-positive cells without or co-localizing with the h-α-Syn transgene, respectively. **d** Dipping analyses revealed significant time-dependent increases in TPH-positive cells expressing h-α-Syn mRNA in the DR and median raphe nucleus (MnR) at different antero-posterior coordinates from bregma (−4.24/-4.48 and −4.48/−4.72 in mm). Likewise, progressive increases of intracellular h-α-Syn mRNA density were found in TPH-positive cells of AAV5-injected mice. **e** Representative coronal midbrain sections showing immunostaining for TPH in raphe nuclei. Scale bar: 200 μm. **f** No differences in the number of TPH-positive cells were found in DR and MnR of vehicle- and AAV5-injected mice. Values are presented as mean ± SEM. *p < 0.05, **p < 0.01, ***p < 0.001 and ****p < 0.0001 compared to vehicle- or AAV5-injected mice. See Supplementary Fig. 1.

We first examined the h-a-Syn transgene expression by injecting AAV5 construct with chicken-β-actin (CBA)-promoter that encodes h-α-Syn (referred to as AAV5) into mouse raphe nuclei. The AAV5 construct was validated in previous studies [44, 46]. To investigate the time-course of h-α-Syn expression, mice were sacrificed at 1, 4, and 8 weeks post-injection. We detected a progressive increase of h-α-Syn mRNA expression in the raphe nuclei compared to control group (*p* < 0.0001), without changes of murine α-Syn mRNA levels (Fig. 1a, b). The maximal increase of h-α-Syn mRNA expression was found at 8 weeks post-injection and reached 290% compared to murine α-Syn mRNA levels (Fig. 1b), reproducing the levels of α-Syn accumulation in patients with duplication or triplication of the gene [13, 14].

A more exhaustive histological analysis was performed in order to evaluate whether the h-α-Syn mRNA co-localized with tryptophan hydroxylase (TPH)-positive cells, specific 5-HT neuronal marker. We found progressively significant increases in the number of TPH-positive cells expressing h-α-Syn mRNA, as well as increases in the intracellular h-α-Syn density in both dorsal raphe (DR) and median raphe (MnR) nuclei (Fig. 1c, d). At 8 weeks post-infusion, ∼84% and 63% of TPH-positive cells expressed the transgene in DR and MnR, respectively, versus ∼60% and 30% detected at 4 weeks post-infusion (*p* < 0.0001). In addition, a variable number of non-TPH-positive cells targeted by the vector were also observed in the raphe nuclei as well as in adjacent brain regions, such as ventrolateral, lateral and dorsolateral periaqueductual gray (Supplementary Fig. 1). Although we did not identify the non-TPH-positive cells, our data indicate that the brain volume transduced by the AAV5 serotype is greater compared to other serotypes, such as 9 or 10 [47], which leaves different populations of cells inside and outside the raphe nuclei expressing the transgene. Next, we did not detect any significant loss of TPH-positive neurons distributed throughout the complete rostro-caudal extent of the raphe nuclei for at least 8 weeks post-infusion (Fig. 1e, f). To rule out any possible effect of the vector itself we performed an experiment with an AAV-EV using exactly the same protocol that we followed for the AAV5 mice. The injection of the AAV-EV did not alter α-Syn or TPH expression, nor did it affect behavioral performance (Supplemental Fig. 2).

**Fig. 2:**
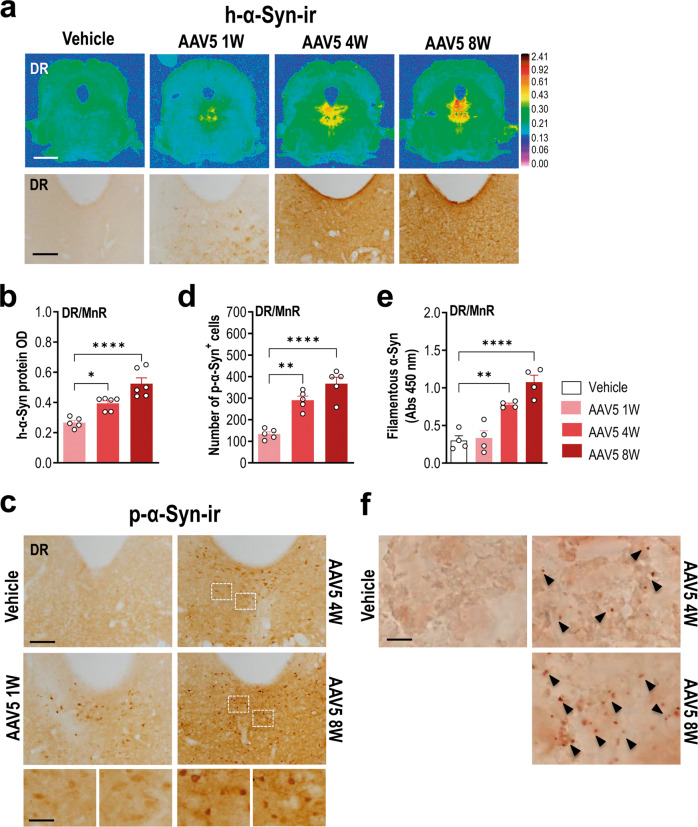
Progressive accumulation of reactive h-α-Syn protein in raphe nuclei. Mice received 1 μl AAV5 construct containing a chicken-β-actin promoter to drive expression of h-α-Syn or vehicle into raphe nuclei and euthanized at 1, 4, and 8 weeks (W) post-injection. **a** Representative coronal midbrain sections showing progressive increases of h-α-Syn protein levels in the raphe nuclei assessed by immunohistochemistry procedures. Top: signal represents the optical density (OD) of autoradiograms as indicated at the right side of the image. Scale bar: 1 mm. Bottom: coronal midbrain sections showing immunostaining for h-α-Syn. Scale bar: 200 μm. **b** Increased h-α-Syn protein levels in AAV5-injected mice. **c** Representative photomicrographs showing gradual increases of phospho-S129-α-Syn (p-α-Syn) levels in the dorsal raphe nucleus (DR) of AAV5-injected mice. Frames indicate areas of highest magnification. Scale bars: 250 μm and 50 μm, respectively. **d** Number of p-α-Syn-positive cells in raphe nuclei. **e** Progressive increase in α-Syn oligomer levels in lysates of raphe nuclei of vehicle- and AAV5-injected mice assessed by ELISA. **f** Representative images of coronal midbrain sections showing specific signal for h-α-syn self-interaction detected by proximity ligand assay (PLA). Scale bar: 10 μm. Black arrowheads show the punctate brown staining likely represents accumulation of aggregated h-α-Syn. Values are presented as mean ± SEM. *p < 0.05, **p < 0.01, ****p < 0.0001 compared to vehicle- or AAV5-injected mice. See Supplementary Fig. 4.

In parallel, we found that AAV5 infusion induced a marked time-dependent increase of h-α-Syn protein density in the raphe nuclei compared to the control group, as assessed by immunohistochemistry (*p* < 0.0001; Fig. 2a,b). The maximum level of h-α-Syn protein reached in raphe nuclei was found at 8 weeks post-injection (∼197% compared to vehicle-infused mice), overlapping with the maximum number of TPH-positive neurons expressing h-α-Syn transgene (Fig. 1c, d).

Phosphorylation of α-Syn at amino-acid serine-129 (p-α-Syn) is a post-transcriptional modification found in ∼90% of α-Syn inclusions in human PD brain tissue and animal models overexpressing α-Syn, frequently used as an indicator for α-Syn aggregation [44, 48, 49]. We, therefore, measured p-α-Syn signal intensity in the raphe nuclei. Our data revealed that AAV5-induced h-α-Syn overexpression leads to strong and progressive phosphorylation of α-Syn in raphe nuclei (Fig. 2c, d). The accumulation of p-α-Syn started early with positive cells being observable already 1-week post-injection, reaching the highest levels at 8 weeks, in close parallelism with h-α-Syn protein accumulation (*p* < 0.0001). Similarly, using ELISA to identify α-Syn aggregation [50, 51], we detected significant increases in filamentous α-Syn levels in raphe nuclei 4 weeks later compared to vehicle-infused mice (*p* < 0.0001; Fig. 2e). We confirmed the α-Syn aggregates in raphe nuclei using the h-α-Syn proximity ligation assay (PLA) which allows detecting proteins in close interaction [52]. While raphe tissue sections from control mice showed no signal, an abundant h-α-Syn puncta signal was found in AAV5-injected mice beginning 4 weeks post-infusion (Fig. 2f).

### Extensive accumulation of h-α-Syn to efferent brain regions

**Fig. 3:**
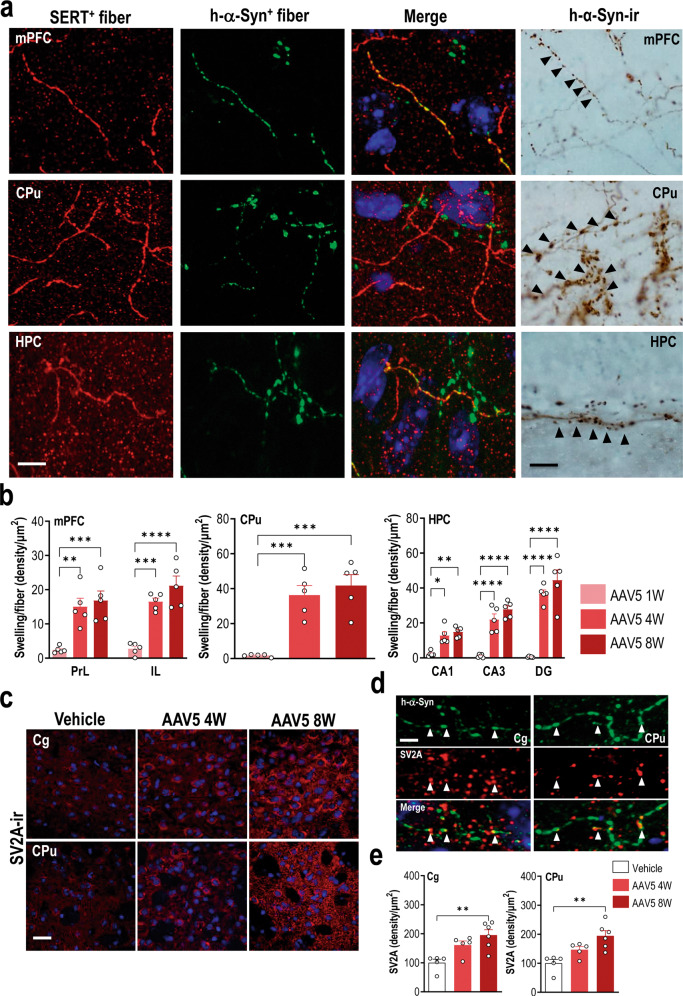
Overexpression of h-α-Syn transgene in raphe 5-HT neurons induces axonal pathology in efferent brain regions. Mice received 1 μl AAV5 construct containing a chicken-β-actin promoter to drive expression of h-α-Syn or vehicle into raphe nuclei and euthanized at 1, 4, and 8 weeks (W) post-injection. **a** Left: representative confocal microscopy images showing SERT and h-α-Syn axonal co-localization in different brain areas including medial prefrontal cortex (mPFC), caudate-putamen (CPu), and hippocampus (HPC) of mice injected with AAV5 examined 4 W later. Most of the h-α-Syn-positive fibers co-stained for SERT, indicating that they come from the raphe nuclei. Scale bar: 25 μm. Right: representative coronal brain sections showing h-α-Syn-positive axonal swellings in mPFC, CPu, and HPC assessed by immunohistochemistry procedure. Axonal swellings are identified with black arrowheads. Scale bar: 25 μm. **b** Progressive increases in the density of axonal swellings were noted due to the accumulation of h-α-Syn protein in the forebrain regions. **c** Representative confocal microscopy images showing punctate immunostaining for SV2A in Cg and CPu. Scale bar: 20 μm. **d** Representative confocal microscopy images of h-α-Syn (green), SV2A (red), and merge illustrating the presynaptic co-localization of the two proteins in fiber swellings. Scale bar: 4 μm. **e** Analysis of SV2A intensity in Cg and CPu showed significant differences between vehicle- and AAV5-treated mice. Values are presented as mean ± SEM. *p < 0.05, **p < 0.01, ***p < 0.001, and ****p < 0.0001 compared to vehicle- or AAV5-injected mice. See Supplementary Fig. 3.

Next, we addressed the question of whether raphe α-Syn pathology could lead to axonal deficits in 5-HT projection brain regions, as previously reported using different PD-like rodent models [44, 53–58]. To investigate the accumulation of h-α-Syn to efferent brain areas, we stained brain sections from vehicle- and AAV5-injected mice, including medial prefrontal cortex (mPFC), cingulate cortex (Cg), caudate-putamen (CPu), and hippocampus (HPC), using antibodies against h-α-Syn and serotonin transporter (SERT). We examined the co-localization of h-α-Syn-positive and SERT-positive fibers at three time points, 1, 4 and 8 weeks later (Fig. 3a, b and Supplementary Fig. 3). Following injection of AAV5 into raphe nuclei, we found an abundant and progressive presence of h-α-Syn-positive fibers from 1-week post-injection (*p* < 0.0001), and subsequently a significant loss of SERT-positive axons was observed in various brain regions innervated by 5-HT neurons 8 weeks later (*p* < 0.05; Supplementary Fig. 3). As previously reported [44], the h-α-Syn signal was solely axonal and no h-α-Syn cell bodies were detected. The highest density of fibers co-localizing h-α-Syn and SERT was seen at 8 weeks post-injection, reaching values of 35.2 ± 2.4%, 31.3 ± 5.7%, 22.9 ± 4.5%, and 19.8 ± 1.7% in CPu > Cg > mPFC > HPC, indicating that the h-α-Syn was anterogradely transported along the axons towards the synaptic terminals. Moreover, at 4 and 8 weeks post-injection, h-α-Syn-positive and SERT-positive fibers had developed a striking distorted appearance with swellings (Fig. 3a, b). Confocal analysis showed an increased accumulation of h-α-Syn protein density in the axonal swellings in all brain areas examined including mPFC, Cg, CPu, and HPC (*p* < 0.001). Notably, the structural axonal changes were accompanied by the presence of PLA-identified h-α-Syn aggregates, suggesting accumulation/aggregation of h-α-Syn in interconnected brain areas could translate into functional axonal deficits (Supplementary Fig. 4).

To determine whether the h-α-Syn accumulation in innervation networks can alter the density of synaptic proteins, such as the synaptic vesicle (SV)-associated proteins, which are ubiquitously expressed in the brain and modulate vesicular function [59], we performed an exploratory analysis of the SV2A protein levels in Cg and CPu from mice overexpressing h-α-Syn in raphe 5-HT neurons. Immunofluorescence analysis showed a progressive increase in punctate SV2A staining in these regions compared to vehicle-treated mice (*p* < 0.01; Fig. 3c, e). Moreover, we identified that this punctate SV2A staining co-localized with h-α-Syn protein in the axonal swellings throughout the Cg and CPu (Fig. 3d), which could lead to altered presynaptic 5-HT function.

### Overexpression of h-α-Syn in 5-HT neurons triggers deficiencies in forebrain 5-HT neurotransmission and BDNF expression and elicits a depressive phenotype

**Fig. 4:**
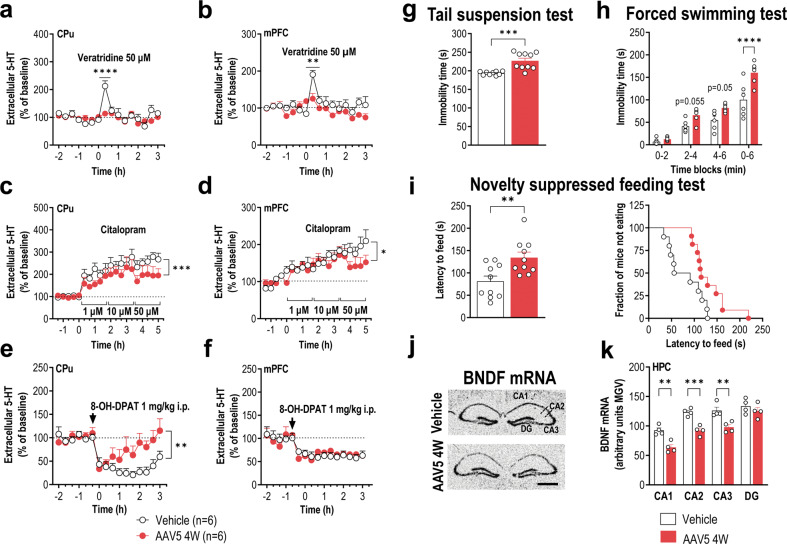
Overexpression of h-α-Syn in the raphe nuclei alters forebrain 5-HT neurotransmission and BDNF mRNA expression, triggering a depressive-like phenotype. Mice received 1 μl AAV5 construct containing a chicken-β-actin promoter to drive expression of h-α-Syn or vehicle into raphe nuclei and were examined at 4 weeks (W) post-injection. At the end of the microdialysis and behavior procedures, the mice were euthanized and transgene expression was confirmed. **a**, **b** Unlike vehicle-injected mice, local veratridine (depolarizing agent, 50 μM) infusion did not induce changes in 5-HT release in the caudate putamen (CPu) and medial prefrontal cortex (mPFC) of AAV5-injected mice. **c**, **d** Local application of citalopram (selective serotonin transporter inhibitor—SERT, 1, 10, and 50 μM) dose-dependently increased the extracellular concentration of 5-HT in CPu and mPFC. This effect was greater vehicle-injected than in AAV5-injected mice. **e**, **f** Systemic administration of 8-OH-DAPT (selective 5-HT_1A_ receptor agonist, 1 mg/kg, intraperitoneal) reduced 5-HT release in CPu, although this effect was lesser in AAV5-injected than in vehicle-injected mice. No differences in mPFC were observed between phenotypes. **g**, **h** AAV5-injected mice evoked a depressive-like state in the tail suspension (**g**) and forced swimming (**h**) tests characterized by a longer immobility time compared to vehicle-injected mice. **i** Likewise, AAV5-injected mice performed worse than vehicle-injected mice in the novelty suppressed feeding test characterized by longer latency to feed. **j** Representative autoradiograms of hippocampal sections of mice showing BDNF mRNA expression. Scale bar: 500 μm. **k** Densitometric analyses showed decreased BDNF mRNA levels in different hippocampal sub-fields of AAV5-injected mice compared to vehicle-injected mice. Values are presented as mean ± SEM. *p < 0.05, **p < 0.01, ***p < 0.001, and ****p < 0.0001 compared to vehicle-injected mice. See Supplementary Fig. 6.

Tissue 5-HT levels were not affected in the raphe nuclei—overlapping with the absence of loss of TPH-positive neurons—as well as in the projection brain areas, with the exception of a reduction in 5-HT levels in CPu and HPC observed at 8 weeks (Supplementary Fig. 5). Therefore, we next performed microdialysis experiments in CPu and mPFC of freely moving mice at 4 weeks post-injection in order to examine whether the axonal h-α-Syn accumulation affects forebrain 5-HT neurotransmission. No differences in baseline extracellular 5-HT concentration were found in both CPu and mPFC between the different groups (Table 1). However, the infusion of the depolarizing agent veratridine (50 μM) by reverse dialysis significantly increased the extracellular 5-HT levels in CPu (*p* < 0.0001) and mPFC (*p* < 0.01) of control mice, but not in mice overexpressing h-α-Syn (Fig. 4a,b), suggesting a marked deficiency in 5-HT reserve pools of AAV5-injected mice.

**Table 1:** Baseline extracellular 5-HT levels in the CPu and mPFC of mice.

Groups	Experimental Conditions	5-HT	
		CPu	mPFC
Vehicle	aCSF + DMSO^a^	6.6 ± 0.7	10.9 ± 0.8
AAV5	aCSF + DMSO^a^	7.1 ± 0.4	15.4 ± 1.3
Vehicle	aCSF^a^	7.9 ± 0.8	11.9 ± 1.2
AAV5	aCSF^a^	10.4 ± 0.9	12.2 ± 0.9
AAV5 + Vehicle	aCSF + DMSO^b^	43.1 ± 9.9	n.e
AAV5 + IND-ASO	aCSF + DMSO^b^	55.2 ± 7.5	n.e
AAV5 + Vehicle	aCSF^b^	19.9 ± 2.4	n.e
AAV5 + IND-ASO	aCSF^b^	15.7 ± 0.8	n.e

Extracellular 5-HT levels are expressed as fmol/20-min fraction. In the experiments involving the evaluation of veratridine effects on extracellular 5-HT levels, DMSO was added in the aCSF. Data are means ± SEM of six mice per group.

^a^Dialysis probe was prepared using the Cuprophan membrane, 6000 Da molecular weight cut-off.

^b^Dialysis probe was provided from CMA (ref.; CMAP000083). For more details, see Supplemental information. n.e. not examined.

We previously showed that changes in endogenous α-Syn levels modify SERT function in cortical and striatal 5-HT terminals of wild-type mice [9]. Local infusion of SERT inhibitor, citalopram (1-10-50 μM), dose-dependently increased the extracellular 5-HT concentration in CPu and mPFC, but, this effect was more pronounced in vehicle- than in AAV5-injected mice (*p* < 0.001; Fig. 4c, d). Furthermore, the systemic administration of 5-HT_1A_ receptor agonist 8-OH-DPAT (1 mg/kg, intraperitoneal) comparably reduced the 5-HT release in mPFC of both phenotypes, but not in CPu. In this region, 8-OH-DPAT effect on 5-HT release was lower in mice injected with AAV5 than with vehicle (*p* < 0.01), suggesting that the inhibitory feedback mechanism mediated by 5-HT_1A_ receptor activation is attenuated by the h-α-Syn overexpression (Fig. 4e, f).

Functional deficits in monoamine neurotransmission, particularly 5-HT, have been extensively associated with anxiety and depression [60, 61], main neuropsychiatric symptoms with a high prevalence in PD patients [29–31]. Previous data showing altered 5-HT neurotransmission provided justification for further analysis, so we conducted several behavioral paradigms routinely used to assess anxiety- and depressive-like behaviors in rodents. AAV5-injected mice showed an increased immobility time in the tail suspension and forced swimming tests compared to vehicle-injected mice (vehicle: 193.5 ± 4.9 s, AAV5: 226.9 ± 7.0 s, *p* < 0.0004 and vehicle: 100.0 ± 5.1 s, AAV5: 160.3 ± 6.9 s, *p* < 0.0004, respectively; Fig. 4g, h). Moreover, AAV5-injected mice showed an increased latency in the novelty suppressed feeding paradigm (*p* < 0.01; Fig. 4i). None of these behavioral changes were driven by changes in the locomotor activity as assessed by the open field test, and comparable anxiety-like behavior was observed in the dark-light box between both groups (Supplementary Fig. 6).

We next examined if the depressive-like phenotype is related to changes in brain-derived neurotrophic factor (BDNF) expression, since several studies support that BDNF dysfunction leads to depression and decreased BDNF levels were observed in the blood and post-mortem brain samples in patients with depression suffering from PD [62–64]. In intra-raphe AAV5 mouse model, we found significant decreases of BDNF mRNA in different HPC regions (*p* < 0.01; Fig. 4j, k), suggesting that α-Syn-induced axonal pathology may be partially caused by defects in synaptic plasticity associated with the insufficient neuronal supply of BDNF, among other neurotrophic factors.

### IND-ASO therapy reduces the h-α-Syn accumulation and alleviates the depressive-like phenotype

**Fig. 5:**
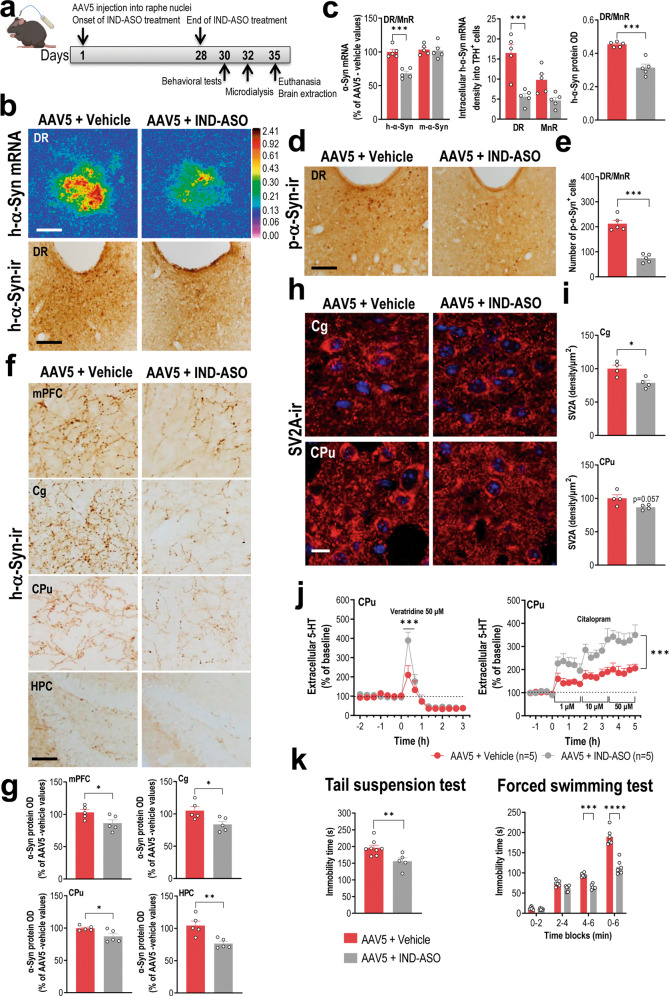
Intracerebroventricular IND-ASO therapy prevents h-α-Syn accumulation and improves 5-HT neurotransmission deficits alleviating the depressive-like phenotype. **a** Treatment schedule. Mice were injected with 1 μl AAV5 construct into raphe nuclei and treated with vehicle or IND-ASO (100 μg/day) into lateral ventricle for 28 days using osmotic minipumps. Mice were euthanized 1 week after completing the treatment. **b** Coronal midbrain sections showing h-α-Syn mRNA (top) and protein (bottom) levels in dorsal raphe nucleus (DR) of AAV5-injected mice and treated with vehicle or IND-ASO assessed by in situ hybridization and immunohistochemistry procedures, respectively. Scale bar: 500 μm. **c** Decreased h-α-Syn mRNA expression, but not murine α-Syn (m-α-Syn), in AAV5-injected mice treated with IND-ASO compared to control group. Likewise, reduced density of h-α-Syn mRNA in TPH-positive cells, as well as h-α-Syn protein level in raphe nuclei was found in AAV5-injected mice treated with IND-ASO compared to those treated with vehicle. **d** Representative images showing immunostaining for phospho-S129-α-Syn (p-α-Syn) in DR of AAV5-injected mice and treated with IND-ASO or vehicle. Scale bars: 250 μm. **e** Significant reduction of the number of p-α-Syn-positive cells in raphe nuclei of AAV5-injected mice treated with IND-ASO compared to those treated with vehicle. **f** Representative coronal brain sections showing h-α-Syn-positive fibers in medial prefrontal cortex (mPFC), cingulate cortex (Cg), caudate-putamen (CPu), and hippocampus (HPC) assessed by immunohistochemistry procedure. Scale bar: 50 μm **g** Analysis of the relative density of h-α-Syn-positive fiber in the different forebrain sections. **h** Representative confocal microscopy images showing punctate immunostaining for SV2A in Cg and CPu of AAV5-injected mice treated with IND-ASO or vehicle. Scale bar: 20 μm. **i** Partial reduction of accumulated SV2A protein levels in AAV5-injected mice treated with IND-ASO compared to those treated with vehicle. **j** Microdialysis approach using different 5-HT agents as in Fig. 4 confirmed an improvement of 5-HT neurotransmission in the CPu of AAV5-injected mice treated with IND-ASO compared to vehicle-treated mice. **k** Treatment with IND-ASO alleviated the depressive-like state of mice in the tail suspension and forced swimming tests. Values are presented as mean ± SEM. *p < 0.05, **p < 0.01, ***p < 0.001, and ****p < 0.0001 compared to AAV5-injected mice treated with vehicle. See Supplementary Fig. 7.

Recently, we designed indatraline-conjugated ASO sequences (1233- and 1337-IND-ASO) that were successfully delivered to monoaminergic neurons in vivo [44, 45]. Both IND-ASOs selectively reduced the accumulation of α-Syn mRNA and protein in aged monkeys and in mouse models overexpressing wild-type or mutant human α-Syn forms in tyrosine-hydroxylase-positive neurons of substantia nigra compacta and locus coeruleus [44, 45]. Therefore, we extended these previous observations and examined the IND-ASO effects (1337 sequence) on the raphe α-synucleinopathy model. For this purpose, AAV5-injected mice into raphe nuclei were treated with vehicle or IND-ASO (100 μg/day, intracerebroventricular) for 4 weeks and euthanized at 7 days after completion of treatment (Fig. 5a). Mice injected with AAV5 and treated with IND-ASO showed a ∼35% reduction in h-α-Syn mRNA expression in raphe 5-HT neurons compared to AAV5-injected mice and treated with vehicle (*p* < 0.001; Fig. 5b, c). Remarkably, IND-ASO treatment did not alter the murine α-Syn mRNA expression or the number of TPH-positive cells (AAV5/IND-ASO: 2158 ± 209; AAV5/Vehicle: 2426 ± 198), which supports the specificity and safety of the employed ASO sequence.

Furthermore, the mice treated with IND-ASO also showed reduced levels of h-α-Syn (∼31%) and p-α-Syn (∼65%) proteins in the raphe nuclei compared to vehicle-treated mice assessed by immunohistochemistry (*p* < 0.001; Fig. 5b–e). Notably, we detected a lower h-α-Syn signal density across different brain regions analysed (mPFC, Cg, CPu, and HPC) after IND-ASO treatment (*p* < 0.05; Fig. 5f, g and Supplementary Fig. 7a). Likewise, a slight reduction was observed in the accumulation of SV2A protein in Cg and CPu of mice injected with AAV5 and treated with IND-ASO compared to AAV5-injected mice treated with vehicle (*p* < 0.05; Fig. 5h, i).

Finally, we found that AAV5-injected/IND-ASO-treated mice showed improved 5-HT neurotransmission in CPu compared to AAV5-injected/vehicle-treated mice assessed by microdialysis procedure (*p* < 0.001; Fig. 5j). In parallel, IND-ASO-treated mice showed increased BDNF mRNA expression in the hippocampus compared to vehicle-treated mice (Supplementary Fig. 7b) and also exhibited better performance in the tail suspension and forced swimming tests with a reduced immobility time (*p* < 0.001; Fig. 5k).

The present data indicate that the α-synucleinopathy in the raphe 5-HT neurons evoked a widespread alteration of 5-HT function and of BDNF expression in HPC. Both effects are likely associated with the depressive-like phenotype, given that the prevention of h-α-Syn overexpression by IND-ASO normalized 5-HT deficits and mouse behavior. Overall, these observations support that α-Syn accumulation in raphe 5-HT neurons partly underlies early non-motor, neuropsychiatric symptoms in PD.

## Discussion

Anxiety and depression are the most relevant neuropsychiatric symptoms that frequently occur in PD, possibly related to serotonergic dysfunctions preceding the loss of dopaminergic neurons, as the disease progresses [24, 27, 33, 37–39]. However, few PD-like animal models have informed on these neuropsychiatric symptoms. In this study, we have developed a novel mouse model of α-synucleinopathy, and demonstrated that AAV5-induced overexpression of h-α-Syn in raphe 5-HT neurons can induce a serotonergic pathology resembling that seen in the PD premotor phase. Our findings showed (i) progressive accumulation, phosphorylation, and aggregation of h-α-Syn in the 5-HT system, (ii) axonal pathology in output regions of raphe neurons, and (iii) 5-HT neurotransmission deficits, which result in a depressive-like phenotype. This mouse model, therefore, provides a potential tool to study the earliest stages of PD pathogenesis, likely involving derangements of the 5-HT system. This model improve our understanding of the emotional brain circuits affected in PD, but is also beneficial for the evaluation of novel disease-modifying therapeutics. Supporting this latter point of view, we show the benefits of novel oligonucleotide-based therapies to decrease α-Syn accumulation selectively in monoaminergic neurons [44, 45, 65], this study].

Using a previously validated AAV5 construct with CBA promoter [44], https://www.michaeljfox.org/research-tools-catalog], we found that 40–80% TPH-positive neurons expressed the h-α-Syn transgene in the raphe nuclei, reaching h-α-Syn mRNA levels that were ∼3-fold the murine phenotype. These ranges reproduce the levels of α-Syn accumulation reported in PD patients with duplications or triplications of *SNCA* gene [13, 14]. However, there are some limitations despite the obvious utility of AAV5 model. Unlike AAV2/6 construct with a cell-specific TPH promoter highly selective for 5-HT neurons [43], a variable number of non-TPH-positive cells located outside the raphe nuclei also expressed h-α-Syn transgene in the AAV5 model. Certainly, several reports indicated that midbrain raphe nuclei contain not only 5-HT neurons but also GABAergic and glutamatergic neurons that do not express TPH and hence do not release 5-HT [66]. Furthermore, populations of peptidergic and DA neurons have also been identified in DR and MR, although the molecular or functional heterogeneity of these neuronal populations has not been determined [67]. In line with this observation, similar findings were reported using an AAV1/2/CBA construct that overexpresses h-α-Syn not only at the locus coeruleus, but also in adjacent regions [68]. Taken together, the most important caveats for the AAV-α-Syn models are the promoter, serotype, and AAV purification and titer because they may determine the volume of transduction and spread of α-Syn expression.

Phosphorylation of α-Syn at amino-acid serine-129 is a dominant pathological modification of α-Syn, since ∼90% of α-Syn is phosphorylated at this position in human Lewy’s bodies, and p-α-Syn signal has been interpreted as the formation of α-Syn aggregates in PD-like animal models [44, 48, 49]. Indeed, genetic and biochemical data suggest that following the elevation of cytosolic α-Syn concentration, the tendency for it is to self-aggregate and form oligomers, and eventually fibrils, leading to structural conformation changes that mediate the toxic effects of α-Syn in cells [55, 69–72]. Hence, aggregation and propagation are α-Syn attributes relevant to its pathogenic role in human synucleinopathies such as PD, among others. In the AAV5 model, we found increases over time in h-α-Syn protein level and serine-129-phosphorylation signal in the raphe nuclei. Importantly, these effects were accompanied by increased immunoreactivity for the filamentous α-Syn forms and PLA-detected h-α-Syn aggregates, which supports the extensive propagation and accumulation of h-α-Syn in output 5-HT brain regions observed in the AAV5 model. Indeed, self-interaction of adjacent h-α-Syn molecules was detected in cortical and subcortical brain regions.

Starting at 4 weeks post-AAV5 injection, boosted immunoreactivity for h-α-Syn was also detected within SERT-positive fibers in the forebrain leading to early axonal pathology, without loss of raphe 5-HT neurons or changes in tissue 5-HT levels, which bears significant implications. In line with these results, a previous study by Wan *et al*., [43] reported similar findings on degenerative changes in axons and dendrites in a rat model overexpressing h-α-Syn in raphe nuclei. Synaptic dysfunction and altered axonal transport has been postulated to be some of the earliest pathological events in PD, in which α-Syn is considered a hub protein by interacting with many synaptic proteins, such as monoamine transporters, cytoskeletal components, chaperones, and several SV-associated proteins [52, 73, 74]. In this regard, increases of the SV2C protein -enriched in the basal ganglia and preferentially localized in DA neurons- were reported in post-mortem PD brain tissue and in mice overexpressing mutant α-Syn [59]. Similarly, we found increased levels of SV2A protein-expressed ubiquitously in the brain- and even co-localizing with h-α-Syn in axonal swellings through CPu and Cg of AAV5 mouse model. Disruptions in SV2A, among others synaptic proteins, e.g. monoamine transporters previously reported [9, 44, 45], may negatively affect 5-HT vesicular function and cause deficits of 5-HT neurotransmission, as early observed in CPu and mPFC at 4 weeks. In addition, mice showed a reduced density of SERT-positive fibers, especially at 8 weeks after AAV5 infusion, when tissue 5-HT content was decreased in some forebrain regions. These above observations emphasize the fact that the deficiency of 5-HT system is not only a result of cell death, but that functionally impaired surviving neurons contribute to the outcome and should be considered as a target for the treatment. Further studies with an additional characterization of the α-Syn interactome at SV-associated proteins are required to confirm serotonergic pathology in the mouse model described herein, highlighting that measurement of serotonergic integrity could be a useful tool to identify individuals at risk to develop PD.

Given the relevance of the 5-HT system for BDNF expression [61, 62, 75], the reduced 5-HT availability may drive the changes in hippocampal BDNF expression described in the AAV5 mouse model. Notably, some studies correlated low circulating levels of BDNF with depression in PD patients [63, 64]. Therefore, h-α-Syn-induced deficits of the 5-HT system, as well as decreased BDNF-mediated plasticity, may be sufficient to evoke a depressive-like behavioral phenotype in the AAV5 mouse model. Despite few preclinical studies, a recent transgenic rat model overexpressing α-Syn showed pronounced changes in the 5-HT system and in parallel, rats elicit an anxiety-like phenotype [42]. Similarly, Wan and colleagues [43], using a rat model, reported that α-Syn overexpression in raphe nuclei combined with overexpression in basal forebrain cholinergic neurons, resulted in pronounced hippocampal neuropathology and significant impairment in the anxious phenotype as assessed in the elevated plus maze, but not in the depressive-like phenotype. Overall, these observations support that the accumulation of α-Syn in the raphe nuclei leads to functional alterations in the brain circuits involved in emotional and mood control in PD-like animal models.

In addition to developing this highly relevant new mouse model of α-synucleinopathy in the 5-HT system that mimics prominent histopathological and neuropsychiatric features of human PD; we have also shown that IND-ASO treatment for four weeks selectively inhibited h-α-Syn synthesis in 5-HT neurons, resulting in less protein accumulation in the forebrain, which in term generates an improvement in 5-HT function and behavioral phenotype. Notably, despite the marked difficulties in delivering oligonucleotides to the brain, there is a growing interest in developing oligonucleotides for brain disorders. In contrast to prior approaches [65, 76], the present study takes advantage to establish an innovative strategy, in which the oligonucleotide was covalently bound to monoamine transporter inhibitor (e.g. IND herein) for its selective delivery to monoaminergic cells, as previously shown using other mouse and monkey models [9, 44, 45, 77]. This strategy allows the possibility of a very precise cellular targeting of α-Syn mRNA, abundantly expressed in monoamine neurons.

In conclusion, we have developed a new mouse model in which overexpression of h-α-Syn in raphe 5-HT neurons mirrors the earliest pathology and behavioral deficits of premotor PD. This opens up new ways to study PD pathogenesis and its treatment, and critically disease-modifying therapies that are likely to have their greatest impact at the onset of the disease process.

## Supplementary information

Supplemental information
