# Supplementary material for: Human α-synuclein overexpression in mouse serotonin neurons triggers a depressive-like phenotype. Rescue by oligonucleotide therapy
Source: Transl Psychiatry. 2022 Feb 24;12:79. doi: 10.1038/s41398-022-01842-z (PMC8873470; doi:10.1038/s41398-022-01842-z)
Supplement: Supplementary file 1 — Supplemental information [file 41398_2022_1842_MOESM1_ESM.docx]

**Supplementary Information for**

**Human α-synuclein overexpression in mouse serotonin neurons triggers a depressive-like phenotype. Rescue by oligonucleotide therapy**

Lluis Miquel-Rio^1,2,3,4# •^ Diana Alarcón-Arís^1,2,3# •^ María Torres-López^1,2 •^ Valentín Cóppola-Segovia^1,5^ ^•^ Rubén Pavia-Collado^1,2,3^ ^•^ Verónica Paz^1,2,3^ ^•^ Esther Ruiz-Bronchal^1,2^ ^•^ Leticia Campa^1,2,3^ ^•^ Carmen Casal^1,2^ ^•^ Andrés Montefeltro^6^ ^•^ Miquel Vila^7,8,9^ ^•^ Francesc Artigas^1,2,3 •^ Raquel Revilla^6 •^ Analia Bortolozzi^1,2,3^

**The PDF file includes:**

- Materials and Methods
- References
- Supplementary Figures S1 – S7
- Supplementary Table S1

**Materials and methods**

**Mice**

Male C57BL/6J mice (14 weeks; *n=*254 for the whole study, Charles River, Lyon, France) were housed under controlled conditions (22 ± 1ºC; 12h light/dark cycle) with food and water available ad-libitum. Animal procedures were conducted in accordance with standard ethical guidelines (EU directive 2010/63 of 22 September 2010) and approved by the local ethical committee (University of Barcelona).

**Mouse model overexpressing h-α-Syn into serotonin (5-HT) neurons**

Recombinant AAV vector serotype 5 (AAV5) with chicken-β-actin (CBA) promoter that encodes the human wild-type α-Syn was used to overexpress h-α-Syn in raphe 5-HT neurons of mice (dorsal raphe nucleus - DR and median raphe nucleus - MnR), as previously reported [44]. The Michael J. Fox Foundation gently provided the AAV5 construct produced and tittered by the UNC Vector Core. We examined any possible effect of the AAV5 vector itself capable of exerting toxicity on 5-HT neurons and causing behavioral alterations using an empty AAV5 serotype vector containing non-coding stuffer DNA (AAV-EV) as a control group. We used this additional control group based on previous studies [44], in which we showed that the AAV5 construct overexpressed the transgene in monoamine neurons, whereas the null viral vector did not induce changes. Isoflurane-anesthetized mice (doses: 4% induction, 2% maintenance) were randomly injected with 1 μl of: i) AAV5 construct (concentration 1.33 x 10^13^ gc/ml), ii) AAV-EV (concentration 1.73 x 10^13^ gc/mL), or iii) vehicle (1PBS: 0.26 mg/ml MgCl_2_, 0.14 mg/ml of KCl in 1xPBS) into raphe nuclei (anterior-posterior AP: -4.5; medial-lateral ML: -1.0; dorsal-ventral DV: -3.2 in mm, relative to bregma with an angle 20º) [78] using a microinjector (KDS-310-PLUS, World Precision Instruments, Sarasota, LF, USA) at 0.4 µl/min rate. The needle was kept in place for an additional 5 min before slowly being withdrawn.

**Conjugated antisense oligonucleotide**

The synthesis and purification of indatraline-conjugated 1337-ASO molecule targeting h-α-Syn (IND-ASO; GenBank accession: NC_000072.6) was performed by nLife therapeutics S.L. (Granada, Spain) as previously reported [9,44,45]. ASO is an 18-mer single stranded DNA molecule with four 2’-O-methyl RNA bases at both ends to protect the internal DNA backbone from nuclease degradation and improve the binding to the target sequence. IND-1337-ASO sequence is [5’-CGCCTTCCACGGTTUUCU-3’]. In brief, ASO synthesis was performed using ultra mild-protected phosphoramidites (Glen Research, Sterling, VA, USA) and H-8 DNA/RNA automatic synthesizer (K&A Laborgeraete GbR, Schaafheim, Germany). Indatraline hydrochloride (IND, triple blocker of monoamine transporters) was conjugated to 5´-carboxy-C10 modified oligonucleotide through an amide bond. This condensation was carried out under organic conditions (DIPEA/DMF, 24 h). Conjugated oligonucleotides were purified by high performance liquid chromatography (HPLC) using a RP-C18 column (4.6 x 150 mm, 5 µm) under a linear gradient condition of acetonitrile. The molecular weight of the oligonucleotide strands was confirmed by MALDI-TOF mass spectrometry (Ultraflex, Bruker Daltonics, Billerica, MA, USA). The concentration of conjugated sequences was calculated on the basis of absorbance at 260 nm wavelength. Stock ASO solutions were prepared in RNAse-free water (Thermo Fisher Scientific, Waltham, MA, USA) and stored at -20ºC until use.

**Intracerebroventricular treatment with an IND-ASO**

For intracerebroventricular IND-ASO treatment, mice were 2% isoflurane-anaesthetized and placed on stereotaxic frame. On the same day that AAV5 construct was injected into raphe nuclei, an osmotic minipump (Alzet model 1004, Durect Corporation, Cupertino, CA, USA) with a cannula (Brain infusion kit 3, Alzet) for perfusion in the lateral ventricle was also implanted from the subcutaneously inserted minipump (AP: -0.34; ML: -1.0; DV: -2.2 in mm, relative to bregma) [78]. Mice received vehicle (2.64 μl/day) or IND-ASO 100 μg/day (16.35 nmol/day) for 28 days. The IND-1337-ASO sequence was extensively characterized in previous studies, and the dose was chosen because it was safe without signs of neuronal and glial toxicity or behavioural changes compared to an IND-conjugated nonsense ASO sequence [9,44,45].

***In situ* hybridization** **(ISH)**

Mice were euthanized by pentobarbital overdose and brains were rapidly removed, frozen on dry ice and stored at -80ºC. Coronal tissue sections containing raphe nuclei and hippocampus (HPC) (14 μm-thick) were obtained and processed, as described elsewhere [9,44,45]. Antisense oligoprobes were complementary to bases: mouse α-Syn (m-α-Syn, 411-447 sequence, GenBank accession NM_001042451), h-α-Syn (sequence 669-709, GenBank accession NM_000345.4), tryptophan hydroxylase - TPH (sequence 360-410, GenBank accession NM_173391), and brain derived neurotrophic factor - BDNF (1188-1238 sequence, GenBank accession NM_007540) (IBA Nucleic Acids Synthesis, Göttingen, Germany). Frozen tissue sections were fixed for 20 min at 4ºC in 4% paraformaldehyde (PFA)/phosphate-buffered saline (1x PBS: 8 mM Na_2_HPO_4_, 1.4 mM KH_2_PO_4_, 136 mM NaCl, and 2.6 mM KCl), washed for 5 min in 3x PBS at room temperature, twice for 5 min each in 1xPBS, and incubated for 2 min at 21ºC in a solution of predigested pronase (Calbiochem, San Diego, CA, USA) at a final concentration of 24 U/mL in 50 mM Tris-HCl, pH 7.5, and 5 mM EDTA. The enzymatic activity was stopped by immersion for 30 s in 2 mg/ml glycine in 1xPBS. Tissues were finally rinsed in 1x PBS and dehydrated through a graded series of ethanol.

Oligonucleotides were individually labeled (2 pmol) at the 3'-end with [^33^P]-dATP (>2500 Ci/mmol; DuPont-NEN, Boston, MA, USA) using terminal deoxynucleotidyl-transferase (TdT, Calbiochem, La Jolla, CA, USA). For hybridization, the radioactively labelled probes were diluted in a solution containing 50% formamide, 4x standard saline citrate, 1x Denhardt’s solution, 10% dextran sulfate, 1% sarkosyl, 20 mM phosphate buffer, pH 7, 250 μg/ml yeast tRNA, and 500 μg/ml salmon sperm DNA. The final concentration of radioactive probes in the hybridization buffer was in the range (~1.5 nM). Tissue sections were covered with hybridization solution containing the labelled probes, overlaid with para-film coverslips and, incubated overnight at 42ºC in humid boxes. Sections were washed four times (45 min each) in a buffer containing 0.6 M NaCl and 10 mM Tris-HCl (pH 7.5) at 60ºC. Hybridized sections were exposed to Biomax-MR film (Kodak, Sigma-Aldrich, Madrid, Spain) for 24-72 h with intensifying screens. For specificity control, adjacent sections were incubated with an excess (50x) of un-labelled probes. Films were analyzed and optical densities (OD) were obtained using MCID image analysis (Mering, Germany). OD was measured in three adjacent sections by a duplicate of each mouse and averaged to obtain individual values. MCID image system was also used to acquire pseudo-color or black and white images, and contrast and brightness of images were the only variables we adjusted digitally.

Additionally, h-α-Syn hybridized midbrain slides were then dipped into Ilford K5 nuclear emulsion (Harman Technology Limited, Cheshire, UK) diluted 1:1 with distilled water. They were exposed in the dark conditions at 4°C for 1 week, and finally developed in Kodak D19 (Kodak, Sigma-Aldrich, Madrid, Spain) for 5 min, and fixed in Ilford Hypam fixer (Harman Technology Limited, Cheshire, UK). Then, tryptophan hydroxylase - TPH (anti-TPH; 1:2000; ref.: AB1541, Sigma-Aldrich, Madrid, Spain) staining was performed in dipped h-α-Syn hybridized slides. Briefly, following endogenous peroxidase inhibition, pre-incubation and incubation were carried out in a 1x PBS/Triton 0.2% solution containing normal serum from secondary antibody host. Primary anti-TPH was incubated 2 days at 4 ºC, followed by incubation with the corresponding biotinylated rabbit anti-sheep (1:500; ref.: BA-6000, Vector Laboratories, Burlingame, CA, USA). The color reaction was performed by incubation with diaminobenzidine tetrahydrochloride solution (DAB, ref.: 5905-50TAB, Sigma-Aldrich, Madrid, Spain). Sections were mounted and embedded in Entellan (Electron Microscopy Sciences, Hatfield, PA, USA). Microphotographs from DR and MnR sections were acquired using a Nikon Eclipse E100 microscope (Nikon, Tokyo, Japan). The number of h-α-Syn mRNA labelling TPH-positive neurons and their intracellular density in midbrain raphe nuclei were assessed in raphe sections corresponding to different antero-posterior levels -4.04 to -4.96 mm from bregma using ImageJ (v1.51s) software. All labelled cells with its nucleus within the counting frame were counted in five consecutive sections and three different microscope fields were analyzed in each section.

**Immunochemistry**

Pentobarbital-anesthetized mice (40 mg/kg, intraperitoneal) were transcardially perfused with 4% PFA in sodium-phosphate buffer (pH 7.4). Brains were extracted, post-fixed 24 h at 4°C in the same solution, and placed in gradient sucrose solution 10–30% for 3 days at 4°C. After cryopreservation, serial 30 μm-thick sections were cut to obtain raphe nuclei, medial prefrontal cortex (mPFC), cingulate cortex (Cg), caudate-putamen (CPu), and HPC. Immunohistochemistry procedure was performed for h-α-Syn (anti-h-α-Syn clone Syn211, 1:1350; ref.: MA1-12874, Thermo Fisher Scientific, Waltham, MA, USA), phospho-S129-α-Syn (anti-phospho-S129-α-Syn 1:2000; ref.: ab51253, Abcam, Cambridge, UK) and TPH (anti-TPH 1:5000; ref.: AB1541, Sigma-Aldrich, Madrid, Spain). Briefly, primary antibodies were incubated overnight at 4ºC, followed by incubation with the corresponding biotinylated anti-mouse IgG1 (1:200; ref.: A-10519, Life Technologies, Carlsbad, CA, USA) for anti-h-α-Syn, biotinylated anti-rabbit IgG (1:200; ref.: BA-1000, Vector Laboratories, Burlingame, CA, USA) for anti-phospho-S129-α-Syn, and biotinylated anti-sheep (1:200; ref.: BA-1000, Vector Laboratories, Burlingame, CA, USA) for anti-TPH according to the manufacturer’s instructions. The color reaction was performed by incubation with DAB solution. Sections were mounted and embedded in Entellan (Electron Microscopy Sciences, Hatfield, PA, USA) and investigated on a Nikon Eclipse E1000 microscope (Nikon, Tokyo, Japan) using 10x, 20x and 60x objectives.

Human α-Syn protein density in raphe nuclei was assessed in regularly spaced 30 μm-thick sections corresponding to different AP levels from -4.04 to -4.96 mm from bregma. Sections were scanned using an Epson high-resolution scanner and sigma scan software was used to compare the optical density in each region of interest. Phospho-S129-α-Syn-positive cells and TPH-positive cells were counted in raphe nuclei using ImageJ (v1.51s) software. All labeled cells with its nucleus within the counting frame were counted in five consecutive sections and three different microscope fields were analyzed in each section. Importantly, TPH-positive neuron counts in the raphe nuclei did not involve the use of stereological procedures. In addition, the intensity of h-α-Syn labelling in mPFC, Cg, CPu, and HPC were quantified in four consecutive sections corresponding to different AP coordinates (mm from bregma): 2.34 to 1.70 for mPFC, 1.42 to 0.02 f or Cg/CPu, and -1.70 to -2.46 for dorsal HPC, respectively, using ImageJ (v1.51s) software. Signal pixel density was obtained by subtracting the background density in the nonspecific condition (without primary antibody).

**Immunofluorescence**

Mice were anaesthetized with pentobarbital and transcardially perfused with 4% PFA in sodium-phosphate buffer (pH 7.4). After cryopreservation, serial 30 μm-thick sections were cut to obtain raphe nuclei, mPFC, Cg, CPu, and HPC. Immunofluorescence procedure was performed for rabbit anti-SV2A (1:250; ref.: PA5-110451, Thermo Fisher Scientific, Waltham, MA, USA). Sections were washed in 1x PBS (pH 7.4) and 1x PBS/Triton 0.2%, and incubated in blocking solution (1x PBS/Triton containing 0.02% gelatin and the corresponding normal serum for the secondary antibody host for 2 hours at room temperature. Primary antibody was incubated overnight at 4ºC, followed by incubation with the secondary antibody donkey anti-rabbit IgG1 Alexa 555 (1:500; ref.: A-31572, Life Technologies, Carlsbad, CA, USA) for 2 hours at room temperature. Finally, nuclei were stained with Hoestch dye (1:10.000; ref.: H3570, Life Technologies, Carlsbad, CA, USA) for 10 min and the sections were mounted in Entellan (Electron Microscopy Sciences, Hatfield, PA, USA).

Tissue sections were imaged using an inverted Nikon Eclipse Ti2-E microscope (Nikon Instruments, Tokyo, Japan) attached to the spinning disk unit Andor Dragonfly 200 (Oxford Instruments Company, Abindong, UK). For all experiments, a Plan Apochromatic 10x, numerical aperture (NA) 0.45 was used. High-precision motorized stage was used to collect the large-scale 3D mosaics of each tissue section. Individual image tiles were 2048 × 2048 pixels with a z-section of 35 µm. For specific regions, we used an oil-immersion objective (Plan Apochromatic Lambda blue 60×, NA 1.4). Samples were excited with 405, 488, and 561 nm laser diodes, respectively. The beam was coupled into a multimode fiber going through the Andor Borealis unit reshaping the beam from a Gaussian profile to a homogenous flat top, and from there it was passed through the 40 µm pinhole disk. Tissue sections were imaged on a high resolution scientific complementary metal oxide semiconductor (sCMOS) camera (Zyla 4.2, 2.0 Andor, Oxford Instruments Company). Fusion software (Andor, Oxford Instruments Company) was used for acquisition and for image processing before analysis. Image stitching and deconvolution were performed using Fusion software (Andor, Oxford Instruments Company). Image analysis was performed with Image J/Fiji open source software (Wayne Rasband, NIH, Bethesda, MD, USA) using ImageJ Macro Language to develop custom Macros. Briefly, maximum projections of the channel of interest (red) were obtained for each section. Region of interest (ROI) was delimited for each brain section so that the maximum area could be measured in all the images acquired. Measurements included area, minimum and maximum gray values and mean gray value. Mean gray values obtained for each image were normalized by the area, and an average value was generated for each animal per ROI.

**Confocal fluorescence microscopy**

Intracellular h-α-Syn distribution in 5-HT axons was examined by confocal microscopy using a Leica TCS SP5 laser scanning confocal microscope (Leica Microsystems Heidelberg GmbH, Manheim, Germany) equipped with a DMI6000 inverted microscope, blue diode (405nm), argon (458/476/488/496/514), diode pumped solid state (561nm) and HeNe (594/633nm) lasers. Brain sections were rinsed with PBS, incubated in 1% bovine serum albumin (BSA, Sigma-Aldrich, Madrid, Spain), PBS/Triton 0.1% and treated with primary antibodies: rabbit-anti-serotonin transporter -SERT- (1:2500; ref.: 24330, Immunostar, Hudson, WI, USA) and mouse-anti-h-α-Syn (1:1350; ref.: MA1-12874, Thermo Fisher Scientific, Waltham, MA, USA). Sections were then incubated at 4ºC overnight, rinsed and treated with secondary antibody A555-anti-rabbit (1:500; ref.: A-31572, Life Technologies, Carlsbad, CA, USA) and A488-anti-mouse IgG1 (1:500; ref.: A-21121, Life Technologies, Carlsbad, CA, USA) for 120 min. Nuclei were stained with Hoescht (1:10.000; ref.: H3570, Life Technologies, Carlsbad, CA, USA). Confocal images were sequentially acquired using 405, 488 and 561 laser lines, AOBS (Acoustic Optical Beam Splitter) as beam splitter and emission detection ranges 415- 480, 500-550 and 571-625 nm, respectively and, the confocal pinhole set at 1 Airy unit. 18-22 images were acquired in stack at 600Hz in a 1024 x 1024-pixel format. Each image average 0.3 μm-thick. Images were analyzed using ImageJ v1.51s software. Co-localization of h-α-Syn- and SERT-positive axons was assessed in several 5-HT projection brain areas including mPFC (prelimbic - PrL and infralimbic – IL cortices), Cg, CPu, and HPC. First, total h-α-Syn (green channel) and SERT (red channel) labelled axonal fiber images were segmented by intensity using the automatic Li’s or Otsu’s [79,80]. Particles smaller than four pixels were discarded from the final resulting images. Second, co-localized h-α-Syn and SERT-positive axonal fibers were selected by intersection of SERT axonal-segmented image and h-α-Syn fiber-segmented image using the Logical AND operation (also known as Boolean AND operation) between two images to generate a new stack with only the axonal signal. Third, h-α-Syn axonal swellings were defined as regions where h-α-Syn shows a high-intensity dot pattern. Swelling regions were segmented from h-a-Syn labelled fiber images using the max entropy intensity auto-threshold method [81]. Finally, area, mean intensity and integrated density were measured from total h-α-Syn-positive fibers, SERT-positive fibers, double h-α-syn- and SERT labelled fibers and h-α-Syn axonal swelling images.

In addition, confocal microscopy studies were also performed to examine the co-localization of h-α-Syn and SV2A in Cg and CPu samples from mice overexpressing h-α-Syn in 5-HT neurons. Rabbit anti-SV2A (1:250; ref.: PA5-110451, Thermo Fisher Scientific, Waltham, MA, USA) and mouse anti-h-α-Syn (anti-h-α-Syn clone Syn211, 1:1000; ref.: MA1-12874, Thermo Fisher Scientific, Waltham, MA, USA) were used as primary antibodies, and donkey anti-rabbit IgG1 Alexa 555 (1:500; ref.: A-31572, Life Technologies, Carlsbad, CA, USA) and goat anti-mouse IgG1 Alexa 488 (1:500; ref: A-21121, Life Technologies, Carlsbad, CA, USA) as secondary antibodies. For the acquisition, an oil-immersion objective (Plan Apochromatic Lambda blue 60x, numerical aperture - NA 1.4) was used. Samples were excited with 405 nm, 488 nm and 561 nm laser diodes, and a single image with a Z-section of 6 µm was acquired for each region of interest.

**ELISA**

Mouse midbrain containing raphe nuclei was dissected out using a Mouse Brain Matrix (Ted Pella, Madrid, Spain), quickly frozen on dry ice and stored at -80ºC. Tissues were lysed by sonication in 50 mM NaCl, 50 mM Tris-HCl, 1% Triton X-100, supplemented with protease (5892970001, Roche, Basel, Switzerland) and phosphatase (A32957, Sigma-Aldrich, Madrid, Spain) inhibitor cocktails, and samples were centrifuged for 10 min at 12.000g at 4ºC. Pellet was re-suspended in same buffer complemented with 2% SDS, and centrifuged for 25 min at 20.000g at 18ºC, and then SDS-soluble proteins were diluted in PBS at a final SDS-concentration of 0,1%. Maxi-Sorp plates (Thermo Nunc Waltham, MA, USA) were with 200 ng of anti-α-Syn filament antibody (MJFR-14-6-4-2 conformation-specific, ref.: ab209538, Abcam, Cambridge, UK) diluted in 50 mM Na_2_CO_3_/NaHCO_3_ buffer, pH 9.6. Micro-titer plate has been pre-coated with an anti-α-Syn clone Syn211 antibody (1:250 ref.: MS-1572, Thermo Fisher Scientific, Waltham, MA, USA). Plates were incubated with the corresponding biotinylated sheep anti-mouse (1:4000, ref.: NXA921, GE Healthcare Life Sciences, Piscataway, NJ, USA). Reaction was stopped adding 50 µl of 1N HCl per well, and absorbance was measured at 450 ± 10 nm in a Multisakan Spectrum reader (Thermo Scientific, Wilmington, USA). The concentration of α-Syn in the samples was determined by comparing the OD of the samples to the standard curve.

**Proximity ligand assay (PLA)**

We used PLA Duolink kits supplied by Sigma-Aldrich (Madrid, Spain) for the detection of h-α-Syn oligomers [52]. Experiments were conducted in accordance with manufacturer’s instructions. To detect human–human α-Syn interactions, anti-h-Syn clone Syn211 (ref.: MA1-12874, Thermo Fisher Scientific, Waltham, MA, USA) was conjugated with Plus or Minus oligonucleotide probes (Duolink® Probemaker kit, Sigma-Aldrich, Madrid, Spain). Briefly, 20 μg of antibody was added to 2 μL of conjugation buffer and the antibody solution was transferred to a vial containing lyophilized oligonucleotides (Plus or Minus). Then, the solution was incubated at room temperature overnight. The conjugates were incubated with 2 μl of stop solution for 30 min at room temperature and suspended in 24 μl of storage solution. Sections containing raphe nuclei and forebrain regions were blocked with Duolink blocking solution and then incubated overnight with PLA-conjugated antibodies probes (1:50 dilution for human–human α-Syn interactions). Sections treated without the Minus probe served as negative controls. On the following day, ligation, amplification and detection were carried out according to manufacturer’s specifications, and the signal was developed with novaRED^TM^ substrates. Sections were mounting with Duolink® in situ bright field mounting medium.

**Intracerebral microdialysis and tissue 5-HT levels**

All reagents used were of analytical grade and were obtained from Merck (Darmstadt, Germany). 5-HT oxalate and 8-OH-DPAT hydrobromide were obtained from Sigma-Aldrich (Madrid, Spain), while citalopram hydrobromide and veratridine were purchased from Tocris (Madrid, Spain). To assess local effects in microdialysis experiments, drugs were dissolved in artificial cerebrospinal fluid (aCSF in mM: NaCl, 125; KCl, 2.5; CaCl_2_, 1.26 and MgCl_2_, 1.18) and administered by reverse dialysis at the stated concentrations (uncorrected for membrane recovery). Veratridine stock solution was prepared in dimethyl sulfoxide (DMSO) and diluted to appropriate concentrations in aCSF to reach 1% DMSO. Citalopram solution was dissolved in aCSF. Concentrated solutions (1mM; pH adjusted to 6.5–7 with NaHCO_3_ when necessary) were stored at -80ºC and working solutions were prepared daily by dilution in aCSF. To evaluate the systemic effects, the drugs were dissolved in saline solution and prepared daily.

Extracellular 5-HT concentration was measured by *in vivo* microdialysis as previously described [9,75]. Briefly, one concentric dialysis probe (Cuprophan membrane; 6000 Da molecular weight cut-off; 1.5 or 2.0 mm-long for CPu or mPFC, respectively) was implanted in CPu (AP, 0.5; ML, -1.7; DV, -4.5 in mm) or mPFC (AP, +2.2; ML, -0.2; DV, -3.4 in mm) [78] of isoflurane-anesthetized mice. Experiments were performed 48 h after surgery in freely moving mice. Additionally, mice overexpressing h-α-Syn and treated with IND-ASO/vehicle were implanted with a guide cannula (CMA 7, ref.: CMAP000138, Harvard Apparatus, Massachusetts, USA) into the CPu (AP, 0.5; ML: -2.4; DV, -2.8 in mm) [78]. A dummy cannula was inserted into guide cannula to prevent contamination and, was secured in place by screwing in onto the guide cannula. The previous day of microdialysis experiments, the dummy cannula was removed and replaced by a 2 mm-long microdialysis probe (CMA 7 microdialysis probe, ref.; CMAP000083, Harvard apparatus, Massachusetts, USA).

5-HT levels in dialysate samples were determined using HPLC coupled to electrochemical detection (+0.7 V, Waters 2465, Cerdanyola, Spain), with 3-fmol detection limit. The mobile phase containing 0.15 M NaH_2_PO_4_.H_2_O, 1.7 mM PICB8, 0.2 mM EDTA and 16 % methanol, adjusted to pH 2.8 with orthophosphoric acid, was pumped at 1 ml/min (Waters 515 HPLC pump). 5-HT was separated on a 2.6 μm particle size C18 column (7.5 x 0.46 cm, Kinetex, Phenomenex, Madrid, Spain) at 28ºC.

In addition, tissue 5-HT content was also determined by HPLC coupled to electrochemical detection. Mice were euthanized at 4 and 8 weeks post-infusion and their brains quickly removed and placed over a cold plate. Midbrain contain raphe nuclei, mPFC, CPu, and HPC from each animal were carefully dissected out using a Rat Brain Matrix (TedPella, Spain), frozen on dry ice, and kept at −80 °C until assayed. Tissues were homogenized in 200 μl of buffer containing 0.4 M perchloric acid, 0.1% sodium metabisulphite, 0.01% EDTA, and 0.1% cysteine and centrifuged at 12,000 × g for 30 min. Aliquots of supernatants were then filtered through 0.45 μm filters (Millex, Spain) and analyzed by HPLC as described above. The mobile phase consisted of 0.1 M KH2PO4, 1mM octyl sodium sulfate, 0.1 mM EDTA (pH 2.65), and 18% methanol.

**Behavioural testing**

Behavioural analyses were performed in mice at 4 weeks post-AAV5 injection with intervals of 1 day between tests. Different behavioural paradigms were used to evaluate anxiety and depressive phenotype. All tests were performed between 10:00 and 15:00 h by an experimenter blind to mouse treatments. On the test day, mice were placed in a dimly illuminated behavioural room and were left undisturbed for at least 1 h before testing [44,45].

*Open field test.* Motor activity was measured in four Plexiglas open field boxes 35x35x40 cm indirectly illuminated (25-40 luxes) to avoid reflection and shadows. The floor of the open field boxes was covered with an interchangeable opaque plastic base that was replaced for each animal. Motor activity was recorded during 15 min by a camera connected to a computer (Videotrack, View Point, Lyon, France). Following variables were measures: horizontal locomotor and exploratory activity, defined as the total distance moved in cm including fast/large (speed > 10.5 cm/s) and slow/short movements (speed 3-10.5 cm/s), and the activation time including mean speed (cm/s) and resting time (s).

*Dark-light box test*. The apparatus consisted of two glass boxes (27 x 21 cm) with an interconnecting grey plastic tunnel (7 x 10 cm). One of these boxes was painted in black, being weakly lit by a red 25-W bulb (42 lux). The other box was lit by a 60-W desk lamp (400 lux) placed 30 cm above the box, which provided the unique laboratory illumination. At the beginning of the test, mice were placed individually in the middle of the dark area facing away from the opening, and were videotaped during 5 min. The following parameters were recorded: a) time spent in the lit box and b) latency of the initial movement from the dark to the lit box. A mouse was considered to enter the new area when all four legs were in this area. The floor of each box was cleaned between the mice.

*Tail suspension test*. Mice were suspended 30 cm above the floor by adhesive tape placed approximately 1 cm from the tip of the tail. Sessions were videotaped for 6 min and the immobility time was measured (Smart, Panlab, Cornella, Spain).

*Forced swim test*. Mice were individually placed into a clear cylinder (15 cm diameter, 30 cm height) containing 20 cm of water maintained at 24–25ºC and the time of immobility was measured for a total of 6 min test and represented in 2-min fractions. The test was recorded using a video camera system (Smart, Panlab, Cornella, Spain).

*Novelty suppressed feeding* *test* is a conflict test that elicits competing motivations: the drive to eat and the fear of venturing into the center of the brightly lit arena. This test was carried out during a 10 min period as previously described [75]. The testing apparatus consisted of a plastic box (35 x 35 x 20 cm), the floor of which was covered with approximately 2 cm of wooden bedding for each animal. 24 h prior to behavioural testing, all food was removed from the home cage. At the time of testing, a single pellet of food was placed on a white paper platform in the center of the box directly illuminated with 1100 lux. An animal was placed in a corner of the box, and a stopwatch was immediately started. The latency to eat (defined as the mouse sitting on its haunches and biting the pellet with the use of forepaws) was timed. Immediately afterwards, the animal was transferred to its home cage and, the latency to feed and the amount of food consumed by the mouse in the subsequent 5 min was measured. Each mouse was weighed before food deprivation and before testing to assess the percentage of body weight loss.

**Statistical analyses**

All values are expressed as the mean ± standard error of the mean (SEM). The number of animals used for each test is reported in the figure legends. Mice infused with the AAV5 vector into raphe nuclei and that did not overexpress the transgene were excluded from the analysis. Statistical comparisons were performed using Graph-Pad Prism 9.01 (GraphPad Prism software, Inc.) using the appropriate statistical tests. Outlier values were identified by the Grubbs’ test (i.e. Extreme Student Zed Deviate, ESD, method) using GraphPad Prism software and excluded from the analysis when applicable. Differences among means were analyzed by either 1- or 2-way analysis of variance (ANOVA) or two-tailed Student's *t*-test as appropriate. When ANOVA showed significant differences, pairwise comparisons between means were subjected to Tukey's post-hoc test or Sidak’s multiple comparisons test as appropriate. Differences were considered significant when *p* < 0.05 (**Supplemental Table 1**).

**References**

1. Franklin K.B.J., Paxinos G. The Mouse Brain in Stereotaxic Coordinates. Academic Press:New York, 2008.
2. Li CH, Tam PKS. An alternative algorithm for minimum cross entropy thresholding. Pattern *Recognit Let* 1998; **19**: 771-776.
3. Otsu N. A threshold selection method from gray-level histograms. *IEEE Trans Syst Man Cybern* 1979; **9**: 62–66.
4. Kapur JN, Sahoo PK, Wong ACK. A new method for gray-level picture thresholding using the entropy of the histogram. *Graph Models Image Process* 1985; **29**: 273–285.

**Supplementary Figures**

**
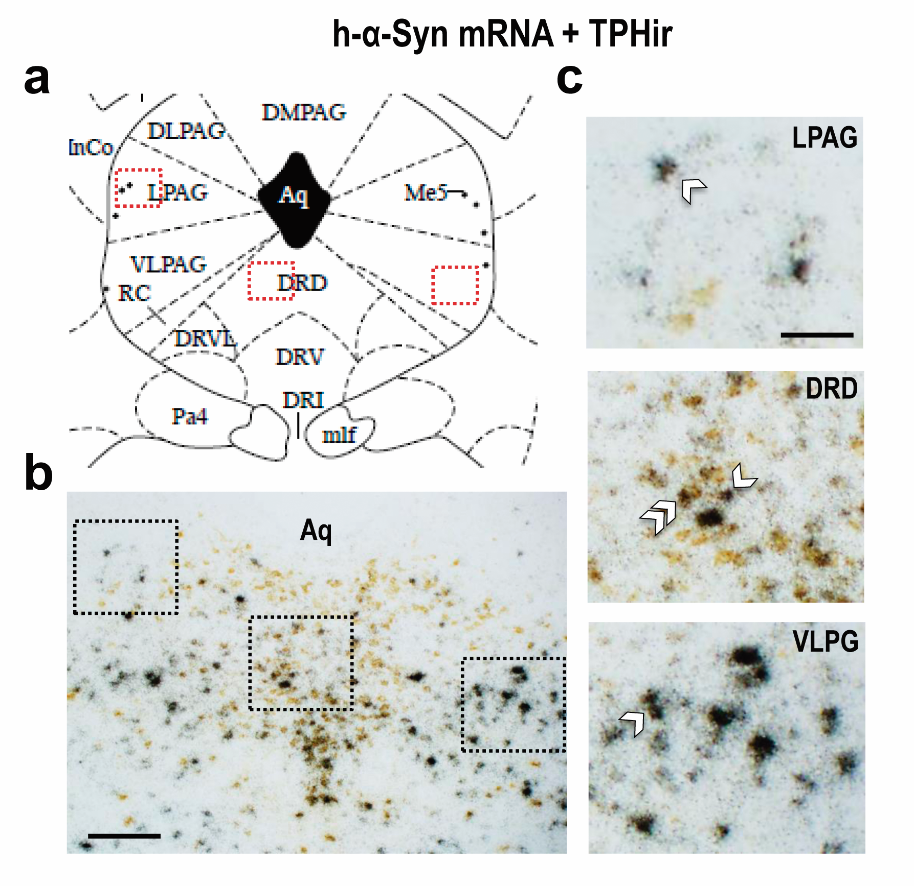
**

**Supplemental Fig. 1** Local injection of AAV5 construct into raphe nuclei also induces h-α-Syn transgene expression in non-TPH cells inside and outside the raphe nuclei. Mice received 1μl AAV5 vector containing a chicken-β-actin promoter to drive expression of h-α-Syn or vehicle into raphe nuclei and euthanized at 1, 4, and 8-weeks (W) post-injection (*n* = 5 mice/group). **a** Schematic coronal representation of mouse midbrain at -4.72 mm (AP coordinate) from bregma [2]. Red frames showing selected brain regions in which h-α-Syn transgene was expressed. **b,c** Photomicrographs showing TPH-positive and non-TPH-positive cells expressing h-α-Syn mRNA (^33^P-oligonucleotide silver grains) in the midbrain of AAV5 mice at 4 W post-injection. Frames indicate areas of highest magnification. Simple and double white arrowheads showing non-TPH-positive and TPH-positive cells co-localizing with the h-α-Syn transcript, respectively. Scale bars: 100 μm (b) and 20 μm (c). Abbreviations: Aq (aqueduct), DRD (dorsal raphe nucleus, dorsal), LPAG (lateral periaqueductual gray), and VLPG (ventral periaqueductual gray). See also Fig. 1.


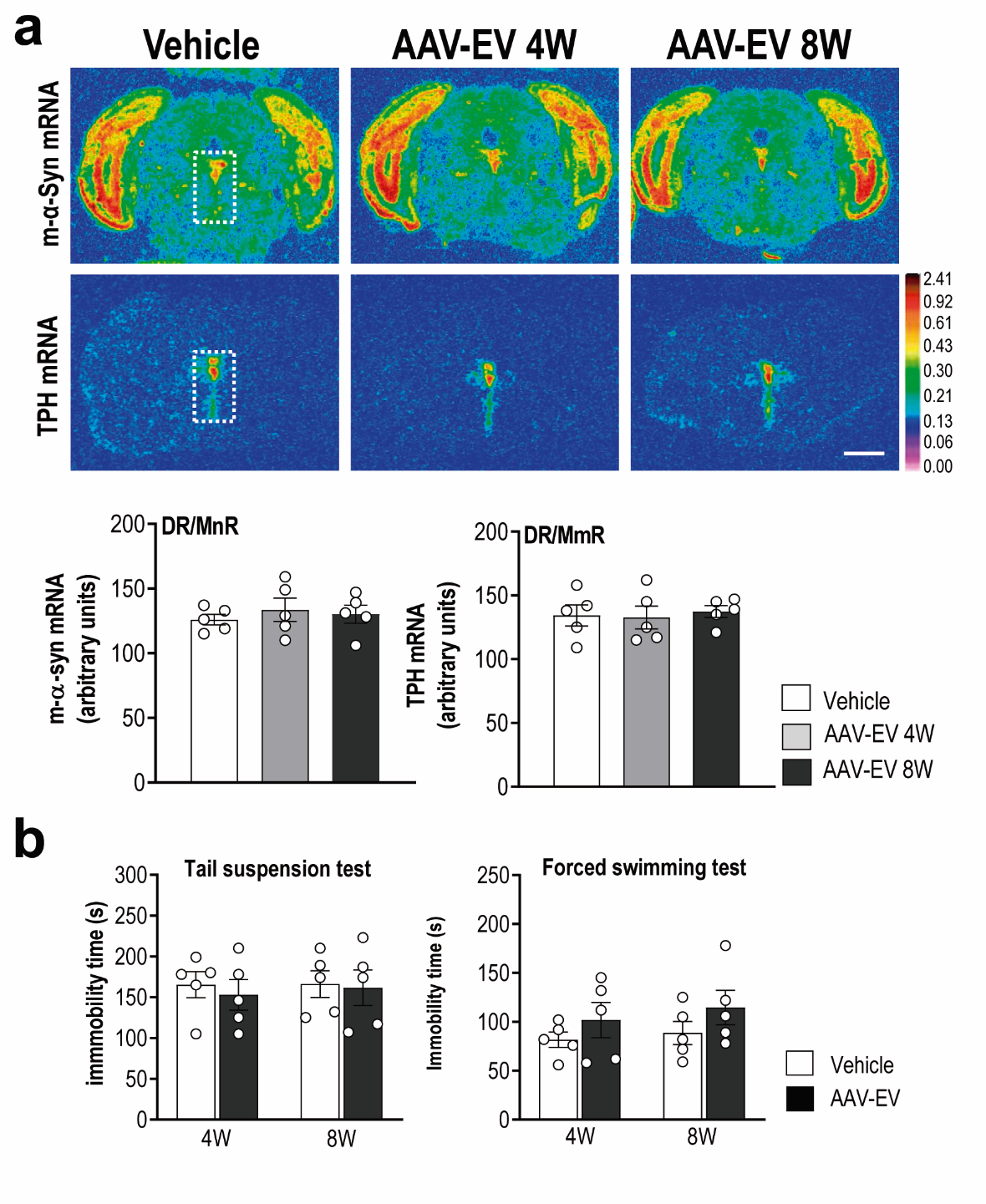


**Supplemental Fig. 2** AAV-empty vector (AAV-EV) injection did not modify mouse α-Syn or TPH mRNA expression, nor did it affect behavioral performance. Mice received 1 μl AAV-EV or vehicle into raphe nuclei and sacrificed at 4 and 8 weeks (W) post-injection (*n* = 5 mice/group). **a** Coronal brain sections showing m-α-Syn and TPH mRNA levels in the raphe nuclei assessed by *in situ* hybridization. Scale bar: 1 mm. Analysis of DR/MnR autoradiograms did not show statistical differences between experimental groups (one-way ANOVA). **b** Mice received vehicle or AAV-EV showed a comparable performance in tail suspension test and forced swimming test (two-way ANOVA). For all figures, data are represented as mean ± SEM.

**
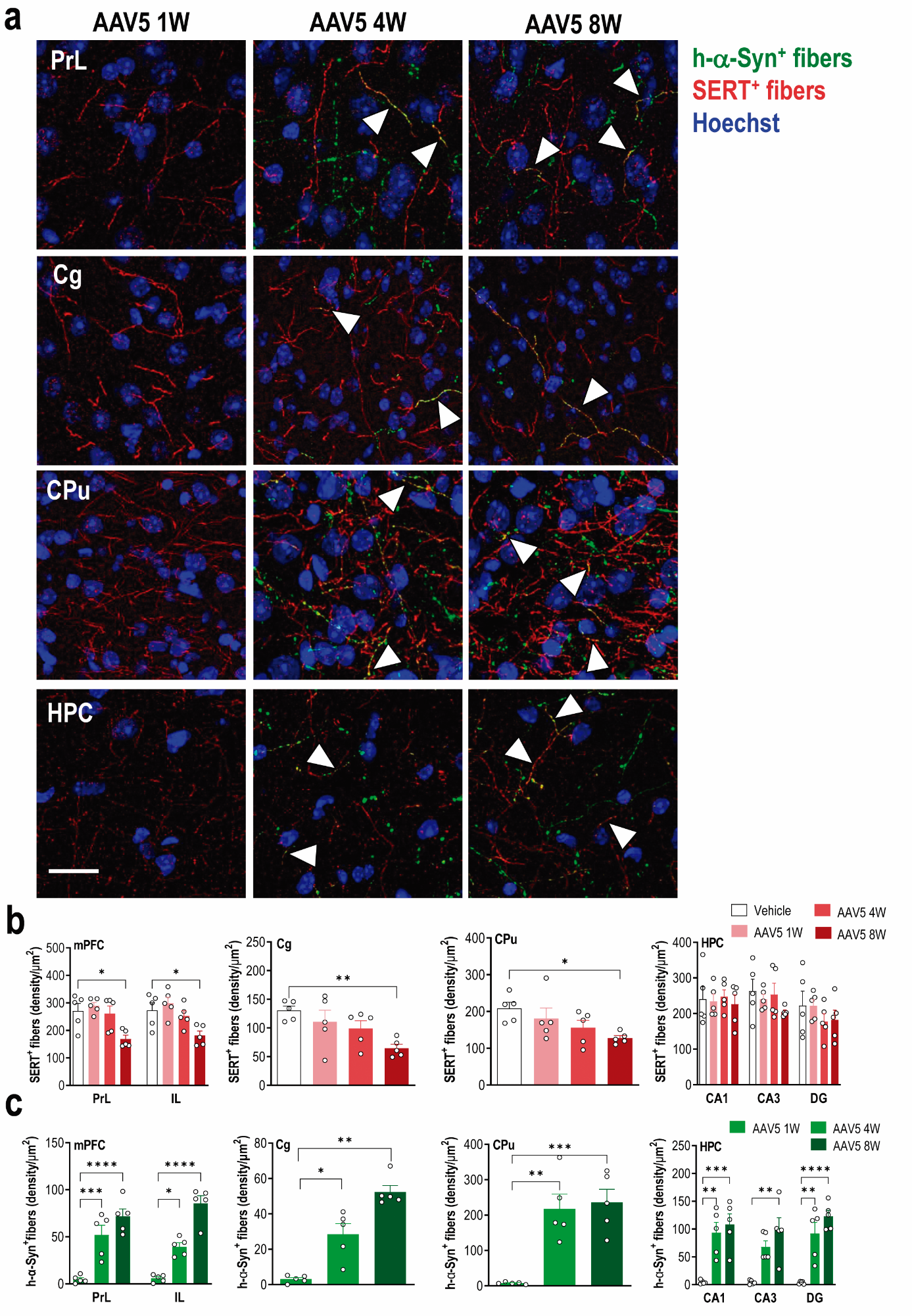
**

**Supplemental Fig. 3** Widespread transport of h-α-Syn protein to 5-HT projection brain areas. Mice received 1 μl AAV5 construct containing a chicken-β-actin promoter to drive expression of h-α-Syn or vehicle into raphe nuclei and euthanized at 1, 4 and 8-weeks (W) post-injection (*n* = 5 mice/group). **a** Representative confocal images stained against serotonin transporter (SERT) and h-α-Syn. White arrowheads show the co-localization of h-α-Syn- and SERT-positive fibers in different brain areas. Scale bar: 25 μm. **b** Bar graphics show the density of SERT-positive fibers in the brain areas analyzed. **c** Bar graphics show progressive increases of h-α-Syn-positive fiber density in different brain areas. Values are presented as mean ± SEM. **p* < 0.05, ***p* < 0.01, ****p* < 0.001 and *****p* < 0.0001 compared to vehicle- or AAV5-injected mice. Abbreviations: PrL (pre-limbic cortex), IL (infra-limbic cortex), medial prefrontal cortex (mPFC), Cg (cingulate cortex), CPu (caudate putamen), and HPC (hippocampus). See also Figure 3.

**
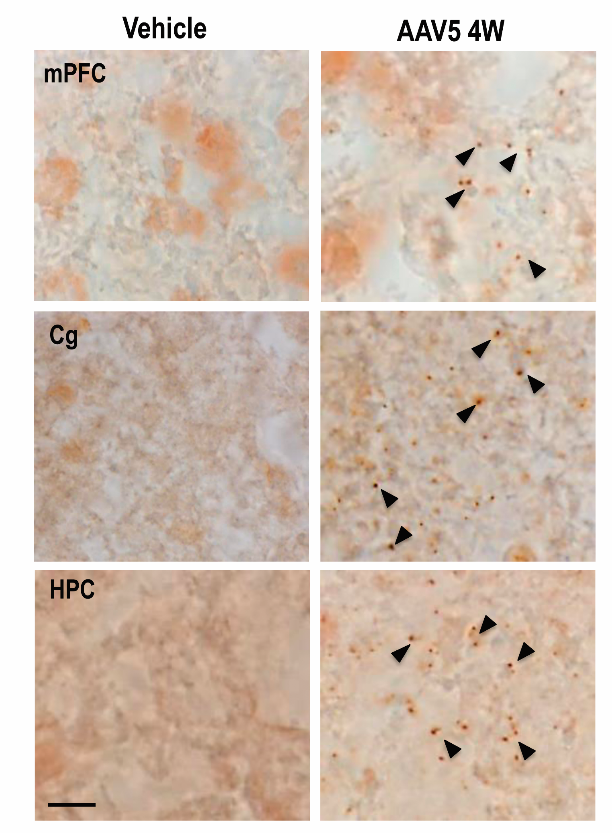
**

**Supplemental Fig. 4** Representative images of coronal brain sections showing specific signal for h-α-syn self-interaction detected by proximity ligand assay (PLA). Mice received 1μl AAV5 construct containing a chicken-β-actin promoter to drive expression of h-α-Syn or vehicle into raphe nuclei and euthanized 4-weeks later (*n* = 5 mice/group). Black arrowheads show the punctate brown staining likely represents accumulation of aggregated h-α-Syn. Scale bar: 10 μm. Abbreviations: medial prefrontal cortex (mPFC), Cg (cingulate cortex), and HPC (hippocampus). See also Figure 2.

**
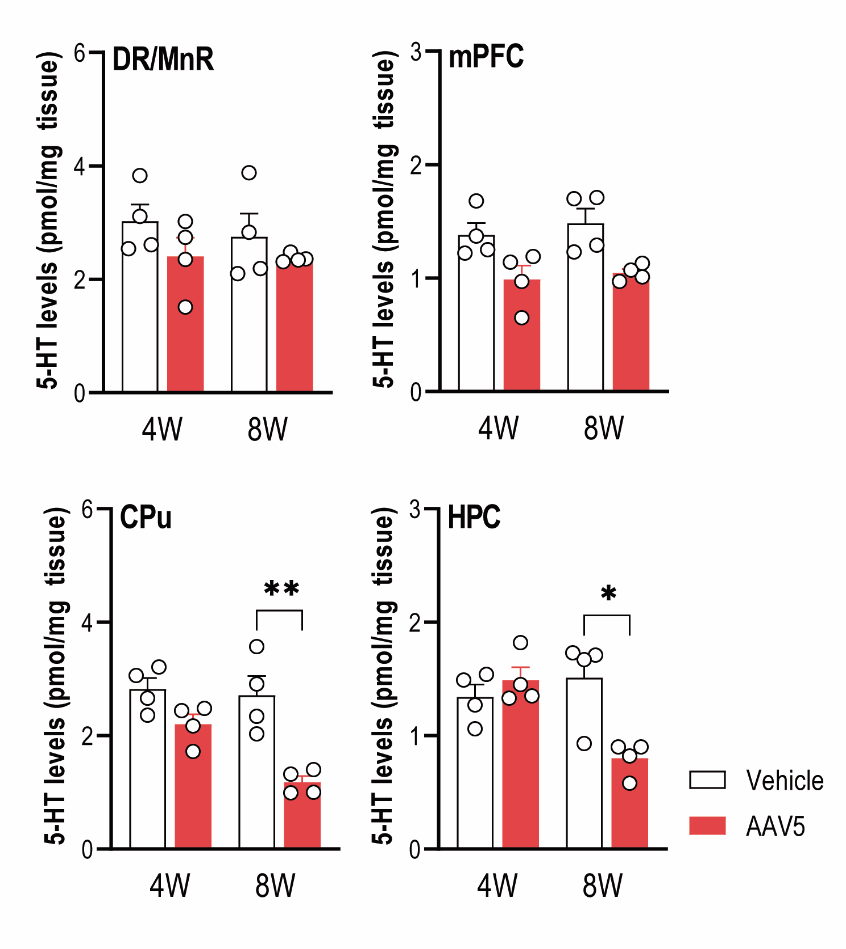
**

**Supplemental Fig. 5** Tissue 5-HT levels in raphe nuclei and forebrain regions. Mice received 1μl AAV5 construct containing a chicken-β-actin promoter to drive expression of h-α-Syn or vehicle into raphe nuclei and tissue 5-HT content was examined 4- and 8-weeks later (*n* = 5 mice/group). Reduced 5-HT content was found in caudate-putamen (CPu) and hippocampus (HPC) at 8-weeks later (**p* < 0.05, ***p* < 0.01 compared to vehicle-injected mice). Data are represented as mean ± SEM.

**
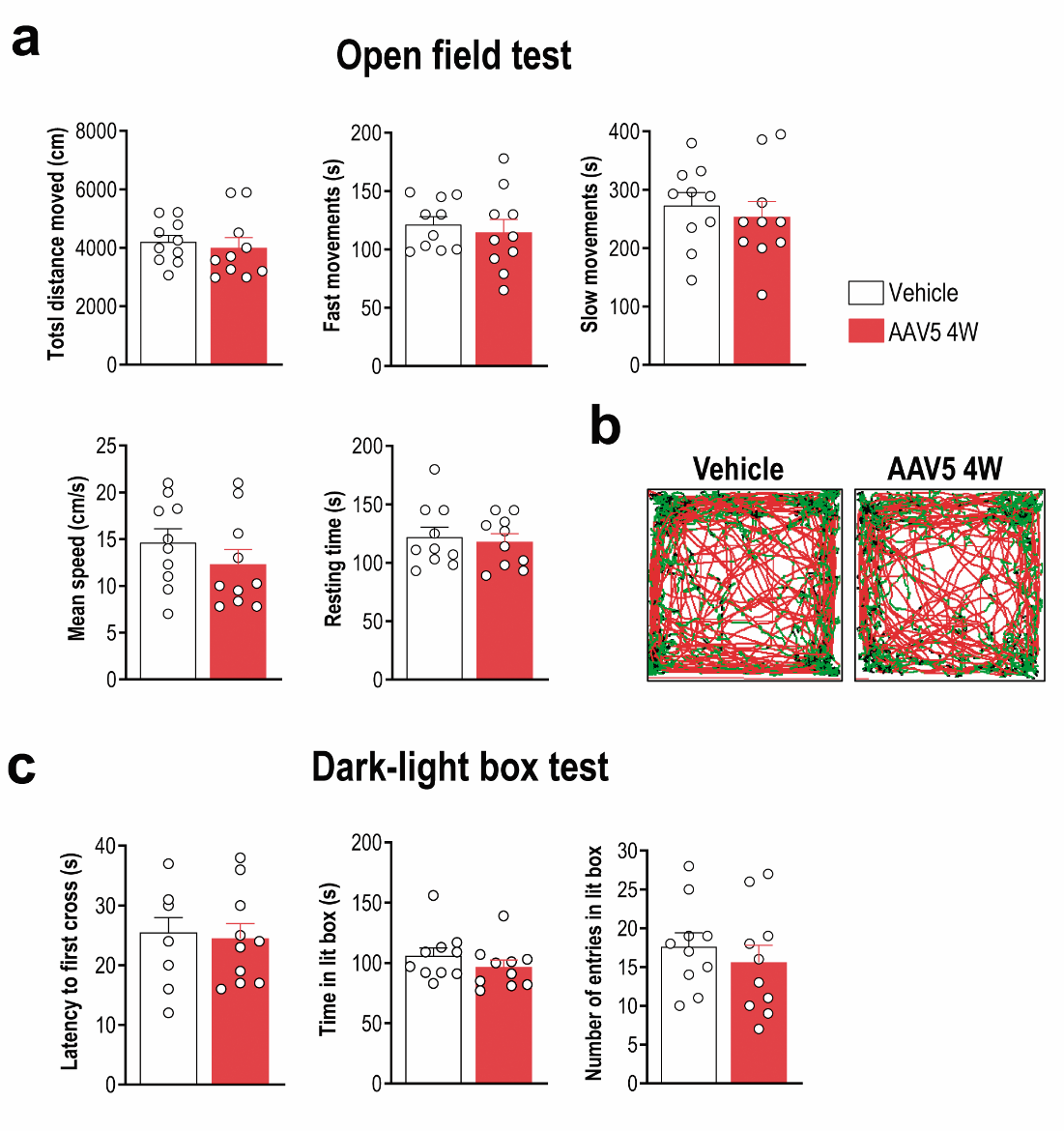
**

**Supplemental Fig. 6** Overexpression of h-α-syn in raphe 5-HT neurons induces a comparable behavioural phenotype to vehicle-injected mice as assessed in the open field test and dark-light box tests. Mice received 1μl AAV5 construct containing a chicken-β-actin promoter to drive expression of h-α-Syn or vehicle into raphe nuclei and tested 4-weeks later (*n* = 10 mice/group). **a** Comparison of spontaneous locomotor activity between AAV5-injected and vehicle-injected mice in the open field test. **b** Representative locomotor activity tracking obtained from both phenotypes. **c** Both phenotypes showed a comparable anxiety-like response in the dark-light box test. Data are represented as mean ± SEM. See also Figure 4.

**
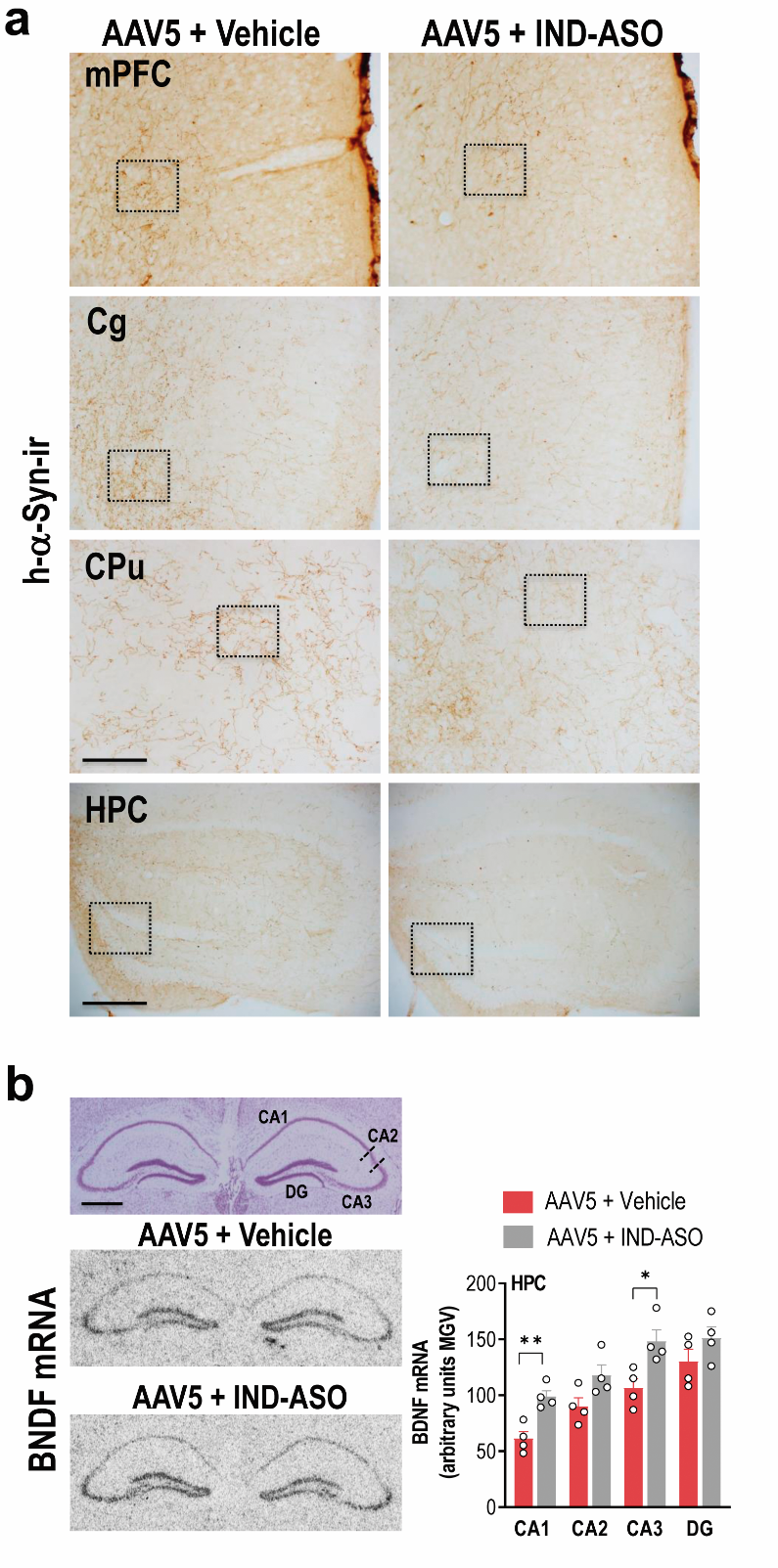
**

**Supplemental Fig. 7** IND-ASO treatment reduces h-α-syn protein levels in forebrain regions and improves hippocampal BDNF levels. See Fig. 5a for the treatment schedule. **a** Representative coronal brain sections showing h-α-syn protein density in different brain areas of AAV5-injected mice treated with IND-ASO or vehicle. Frames showing the enlarged brain region in Fig. 5e. Scale bar: 50 μm (mPFC, Cg, and CPu) and 100 μm (HPC). **b** Representative autoradiograms of hippocampal sections of mice showing BDNF mRNA expression. Scale bar: 500 μm. Densitometric analyses were performed in different hippocampal regions: CA1, CA2, CA3 and DG shown in the cresyl violet-stained section (top). Levels of BDNF mRNA are shown in the bar graphs next to the representative autoradiograms (*n* = 4 mice/group). Data are represented as mean ± SEM. **p* < 0.05, ***p* < 0.01 compared to AAV5-injected mice treated with vehicle. Abbreviations: medial prefrontal cortex (mPFC), Cg (cingulate cortex), CPu (caudate-putamen), and HPC (hippocampus).

**Supplemental Table 1. Statistical analyses**

| **Procedure** | **Measurement** | **Statistical test** | **Comparison** | **Statistics** | **Degrees of freedom** | **p** | **Figure** |
| --- | --- | --- | --- | --- | --- | --- | --- |
| *In situ* hybridization | h/m-α-syn mRNA | 2way ANOVA | F1 α-syn form  F2 Group  Interaction | F=187.6  F=37.61  F=41.48 | 1, 30  3, 30  3, 30 | 0.0001***  0.0001***  0.0001*** | 1b |
|  |  |  |  |  |  |  |  |
| Immuno-histochemistry | Co-localization TPH+ cells/h-α-syn mRNA  Intracellular h-α-syn density | 2way ANOVA | F1 Brain area  F2 Group  Interaction  F1 Brain area  F2 Group  Interaction | F=19.66  F=62.79  F=2.024  F=0.004  F=48.51  F=0.002 | 3, 26  1, 26  3, 26  1, 13  1, 13  1, 13 | 0.0001***  0.0001***  0.1352  0.9634  0.0001***  0.9634 | 1d |
|  |  |  |  |  |  |  |  |
| Immuno-histochemistry | h-α-syn protein (DR/MnR) | 1way ANOVA | F Group | F=22.65 | 2, 14 | 0.0001*** | 2b |
|  |  |  |  |  |  |  |  |
| Immuno-histochemistry | p-α-syn protein (DR/MnR) | 1way ANOVA | F Group | F=31.20 | 2, 12 | 0.0001*** | 2d |
|  |  |  |  |  |  |  |  |
| Elisa | Filamentous α-syn | 1way ANOVA | F Group | F=24.37 | 3, 12 | 0.0001*** | 2e |
|  |  |  |  |  |  |  |  |
| Confocal microscopy | Swelling/fiber  (PrL and IL)  Swelling/fiber  (CPu)  Swelling/fiber  (HPC) | 1/2way ANOVA | F1 Brain area  F2 Time  Interaction  F Group  F1 Brain area  F2 Time  Interaction | F=1.55  F=37.61  F=0.54  F=20.57  F=32.46  F=93.35  F=10.17 | 1, 24  2, 24  2, 24  2, 12  2, 36  2, 36  4, 36 | 0.2246  0.0001***  0.5895  0.0001***  0.0001***  0.0001***  0.0001*** | 3b |
|  |  |  |  |  |  |  |  |
| Immunofluorescence | SV2A  (Cg)  SV2A  (CPu) | 1way ANOVA | F Group | F=9.228  F=10.35 | 2, 13  2, 13 | 0.0032**  0.0020** | 3e |
|  |  |  |  |  |  |  |  |
| Microdyalisis | 5-HT levels  (veratridine effect in CPu)  5-HT levels  (veratridine effect in mPFC)  5-HT levels  (citalopram effect in CPu)  5-HT levels  (citalopram effect in mPFC)  5-HT levels  (8-OHDPAT effect in CPu)  5-HT levels  (8-OHDPAT effect in mPFC) | 2way ANOVA | F1 Group  F2 Time  Interaction  F1 Group  F2 Time  Interaction  F1 Group  F2 Time  Interaction  F1 Group  F2 Time  Interaction  F1 Group  F2 Time  Interaction  F1 Group  F2 Time  Interaction | F=1.774  F=8.758  F=4.368  F=7.024  F=4.264  F=1.884  F=17.18  F=8.171  F=0.537  F=6.298  F=6.105  F=0.993  F=7.731  F=8.974  F=2.259  F=0.062  F=8.566  F=0.512 | 1, 9  15, 135  15, 135  1, 7  15, 105  15, 105  1, 320  19, 320  19, 320  1, 200  19, 200  19, 200  1, 150  15, 150  15, 150  1, 160  15, 160  15, 160 | 0.22  0·001***  0·001***  0.03*  0.0001***  0.03*  0.001***  0.001***  0.94  0.01*  0.001***  0.47  0.0194*  0.0001***  0.0068***  0.8023  0.0001***  0.9315 | 4a  4b  4c  4d  4e  4f |
|  |  |  |  |  |  |  |  |
| Behaviour | Tail suspension test  Forced swimming test  Novelty suppressed feeding test | *t*-test  2way ANOVA  *t*-test | Group  F1 Group  F2 Time  Interaction  Group | T=4.414  F=27.67  F=79.33  F=4.140  T=3.092 | df=17  1, 40  3, 40  3, 40  df=18 | 0.0004***  0.0001***  0.0001***  0.0120*  0.0063** | 4g  4h  4i |
|  |  |  |  |  |  |  |  |
| *In situ* hybridization | BDNF mRNA | 2way ANOVA | F1 Group  F2 Brain area  Interaction | F=46.53  F=36.82  F=2.356 | 1, 24  3, 24  3, 24 | 0.0001***  0.0001***  0.0971 | 4k |
|  |  |  |  |  |  |  |  |
| *In situ* hybridization/Immunohistochemistry | h-α-syn mRNA  (DR/MnR)  Intracellular h-α-syn mRNA/TPH+ cells  h-α-syn protein  (DR/MnR) | *t*-test  1way ANOVA  *t*-test | Group  F Group  Group | T=6.456  F=14.62  T=6.145 | df=8  3, 16  df=8 | 0.0002***  0.0001***  0.0003*** | 5c |
|  |  |  |  |  |  |  |  |
| Immuno-histochemistry | p-α-syn protein (DR/MnR) | *t*-test | Group | T=8.993 | df=8 | 0.0001*** | 5e |
|  |  |  |  |  |  |  |  |
| Immuno-histochemistry | h-α-syn protein  (mPFC)  h-α-syn protein  (Cg)  h-α-syn protein  (CPu)  h-α-syn protein  (HPC) | *t*-test | Group | T=2.437  T=2.753  T=2.721  T=3.690 | df=8  df=8  df=8  df=8 | 0.05*  0.05*  0.0262*  0.0061** | 5g |
|  |  |  |  |  |  |  |  |
| Immunofluorescence | SV2A  (Cg)  SV2A  (CPu) | *t*-test | Group | T=3.221  T=2.349 | df=6  df=6 | 0.0181*  0.0571 | 5i |
|  |  |  |  |  |  |  |  |
| Microdyalisis | 5-HT levels  (veratridine effect in CPu)  5-HT levels  (citalopram effect in CPu) | 2way ANOVA | F1 Group  F2 Time  Interaction  F1 Group  F2 Time  Interaction | F=8.186  F=31.84  F=3.801  F=114.5  F=14.66  F=2.615 | 1, 159  15, 159  15, 159  1, 160  19, 160  19, 160 | 0.0048**  0·0001***  0·0001**  0.0001***  0.0001***  0.0006** | 5j |
|  |  |  |  |  |  |  |  |
| Behaviour | Tail suspension test  Forced swimming test | *t*-test  2way ANOVA | Group  F1 Group  F2 Time  Interaction | T=3.059  F=92.90  F=356.1  F=27.03 | df=11  1, 40  3, 40  3, 40 | 0.0109*  0.0001***  0.0001***  0.0001*** | 5k |
|  |  |  |  |  |  |  |  |
| Confocal microscopy | SERT fibers  (PrL and IL)  SERT fibers  (Cg)  SERT fibers  (CPu)  SERT fibers  (HPC)  h-α-syn fibers  (Prl and IL)  h-α-syn fibers  (Cg)  h-α-syn fibers (CPu)  h-α-syn fibers  (HPC) | 2way ANOVA  1way ANOVA  1way ANOVA  2way ANOVA  2way ANOVA  1way ANOVA  1way ANOVA  2way ANOVA | F1 Brain area  F2 Time  Interaction  F Group  F Group  F1 Brain area  F2 Time  Interaction  F1 Brain area  F2 Time  Interaction  F Group  F Group  F1 Brain area  F2 Time  Interaction | F=0.0066  F=11.68  F=0.098  F=4.448  F=3.069  F=2.787  F=1.123  F=0.4255  F=0.020  F=62.74  F=2.043  F=36.12  F=15.61  F=1.038  F=45.38  F=0.3710 | 1, 32  3, 32  3, 32  3, 16  3, 16  2, 48  3, 48  6, 48  1, 24  2, 24  2, 24  2, 12  2, 12  2, 36  2, 36  4, 36 | 0.7982  0.0001***  0.9605  0.0187*  0.05*  0.0716  0.3491  0.8583  0.8887  0.0001***  0.1516  0.0001***  0.0005***  0.3645  0·0001***  0.8277 | Suppl Figure 3b  Suppl Figure 3c |
|  |  |  |  |  |  |  |  |
| Tissue 5-HT levels | Tissue 5-HT levels  (CPu)  Tissue 5-HT levels  (HPC) | 2way ANOVA | F1 Group  F2 Time  Interaction  F1 Group  F2 Time  Interaction | F=23.92  F=6.636  F=4.313  F=4.627  F=3.916  F=10.75 | 1, 12  1, 12  1, 12  1, 12  1, 12  1, 12 | 0.001**  0.05*  0.06  0.05  0.07  0.0066** | Suppl Figure 5 |
|  |  |  |  |  |  |  |  |
| *In situ* hybridization | BDNF mRNA | 2way ANOVA | F1 Group  F2 Brain area  Interaction | F=26.17  F=18.36  F=0.5667 | 1, 24  3, 24  3, 24 | 0.0001***  0.0001***  0.6423 | Suppl Figure 7b |
